# High-Affinity Functional Fluorescent Ligands for Human β-Adrenoceptors

**DOI:** 10.1038/s41598-017-12468-3

**Published:** 2017-09-26

**Authors:** Gyuzel Y. Mitronova, Gražvydas Lukinavičius, Alexey N. Butkevich, Tobias Kohl, Vladimir N. Belov, Stephan E. Lehnart, Stefan W. Hell

**Affiliations:** 1 Department of NanoBiophotonics, Max Planck Institute for Biophysical Chemistry, Am Fassberg 11, 37077 Göttingen, Germany; 2 Heart Research Center Göttingen, Department of Cardiology & Pulmonology, University Medical Center Göttingen, Robert-Koch-Str 40, 37075 Göttingen, Germany; 3 German Center for Cardiovascular Research (DZHK), partner site Göttingen, Germany

## Abstract

Visualization of the G-protein coupled receptor (GPCR) is of great importance for studying its function in a native cell. We have synthesized a series of red-emitting fluorescent probes targeting β-adrenergic receptor (βAR) that are compatible with confocal and Stimulated Emission Depletion (STED) microscopy as well as with Time-Resolved Fluorescence Resonance Energy Transfer (TR-FRET) binding assay in living cells. The probe based on the agonist BI-167107 and fluorescent dye KK114 demonstrates nanomolar binding affinity and up to nine-fold β_2_AR selectivity over β_1_AR. Carazolol-derived probes are fluorogenic and allow no-wash imaging experiments. STED microscopy of β_2_ARs stained at the native expression level on pancreatic CAPAN cells provides two-fold improvement in lateral optical resolution over confocal mode and reveals the formation of receptor microdomains. These probes retain their functional (agonist or antagonist) properties, allowing simultaneous modulation of cyclic adenosine monophosphate (cAMP) levels and receptor internalization as well as imaging receptor localization.

## Introduction

The β adrenergic receptor (β-adrenoceptor, βAR) is an important model system of the ternary signaling complex with the stimulatory G protein (Gs) activating adenylyl cyclase and Ca^2+^ channels and is relevant for human physiology and disease^1,2^. Because βAR-ligand complexes are nanoscale sized and highly dynamic, complex methods are required to determine ligand binding –parameters and internalization kinetics^3^. Fluorescence microscopy directly retrieves important spatiotemporal information about receptor-ligand and receptor-receptor interactions^4,5^. Recent developments in superresolution microscopy enable molecular imaging at nanometer resolution in live cells^6–10^. For this purpose, the availability of high-affinity fluorescent βAR ligands is a prerequisite. Several factors can negatively affect the imaging results, namely: inadvertent loss of ligand affinity to G-protein coupled receptors (GPCRs) and/or quenching of the fluorescence upon dye-ligand conjugation, excessive environmental sensitivity of the probe and unspecific binding^4,11^.

**Figure 1:**
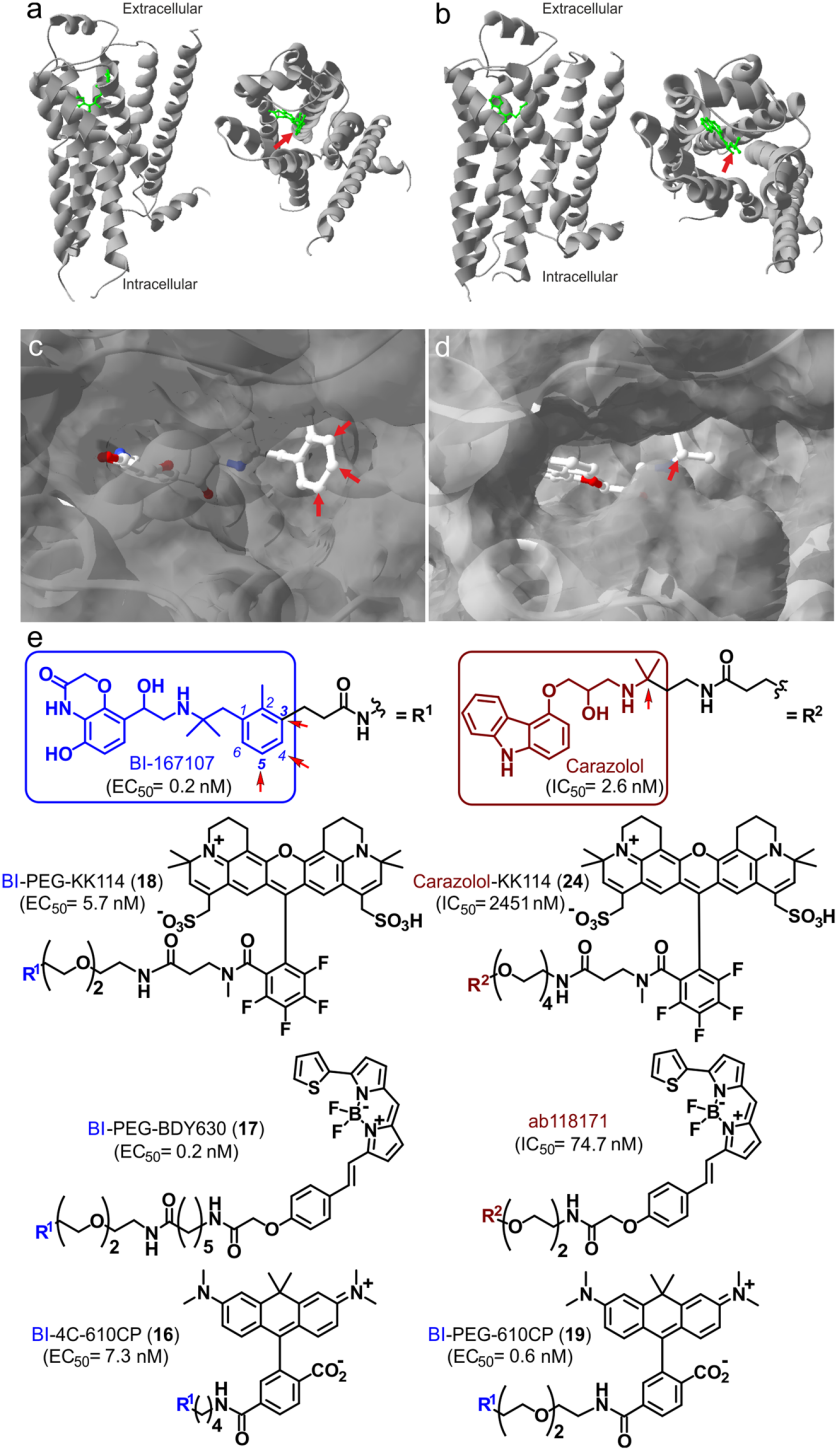
β_2_AR binding sites and chemical structures of fluorescent derivatives of BI-167107 and carazolol. (**a**,**b**) Side view and extracellular view of (*R*)-enantiomer BI-167107 (**a**, PDB ID code 4LDE^14^) and (*S*)-carazolol (**b**, PDB ID code 5JQH^15^) ligands (green) bound to the β_2_AR (gray). T4 lysozyme and nanobody are omitted for clarity. (**c**,**d**) Magnified view of BI-167107 (**c**) and carazolol (**d**) within the binding pocket of the receptor. (**e**) Chemical structures of fluorescent derivatives of BI-167107 (with EC_50_ values) and carazolol (with IC_50_ values). Red arrows indicate positions suitable for attachment of fluorescent reporters.

We present here several novel fluorescent βAR ligands based on the antagonist carazolol^12^ and on the recently reported high-affinity agonist BI-167107^12,13^. Compared to other βAR ligands, BI-167107 and carazolol display nanomolar affinities and slow off-rates^12^. The dissociation constants (*K*_*d*_) for both ligands lie in the subnanomolar range (0.084 nM for BI-167107; 0.135 nM for carazolol)^12^. We hypothesized that conjugation of a fluorophore to BI-167107 or carazolol would provide fluorescent βAR ligands retaining high affinities and sufficiently slow dissociation rates to be useable as probes for optical microscopy. The co-crystal structures reported for β_2_AR complexes with (*R*)-enantiomer of BI-167107 and (*S*)-carazolol^12,14,15^ suggest the possible attachment sites of the fluorescent reporter inflicting minimal alterations to the receptor-ligand interaction surface (Fig. 1a–d). Accordingly, we synthesized fluorescent probes based on the selected ligands, BI-167107 and carazolol, and red-emitting fluorophores (λ_em_ > 600 nm) BODIPY 630/650-X^11^, KK114^16^ and 610CP^17^.

## Results

### Synthesis of BI-167107 derivatives

To the best of our knowledge, there have been no published reports on fluorescent analogs of BI-167107, wich is a 5-hydroxy-4*H*-benzo[1,4]oxazin-3-one derivative with an ethanolamine residue (a catecholamine mimic) linked to a 1,2,3-trisubstituted phenyl ring via the amino group (Fig. 1e). Additional substitution in the phenyl ring and its influence on the potency and efficacy towards the β_1_- and β_2_-adrenoceptors has been studied^13^ with the racemic derivatives being full or even super agonists of the β_2_AR. In particular, racemic BI-167107, having a 2-methyl substituent in the phenyl ring, was classified as a full agonist of the β-adrenoceptors and demonstrated six-fold β_2_AR selectivity over β_1_AR. The analog with a carboxylic acid group at the C-3 position of the phenyl ring displayed even higher β_2_ subtype selectivity^13^. Herein we report the results obtained with the racemic derivatives of BI-167107. Our goal was to synthesize fluorescent βAR probes that are applicable in optical microscopy and we focused on the preparation of BI-167107 derivatives with various linkers and fluorescent ligands attached to the position 3 of the phenyl ring.

**Figure 2:**
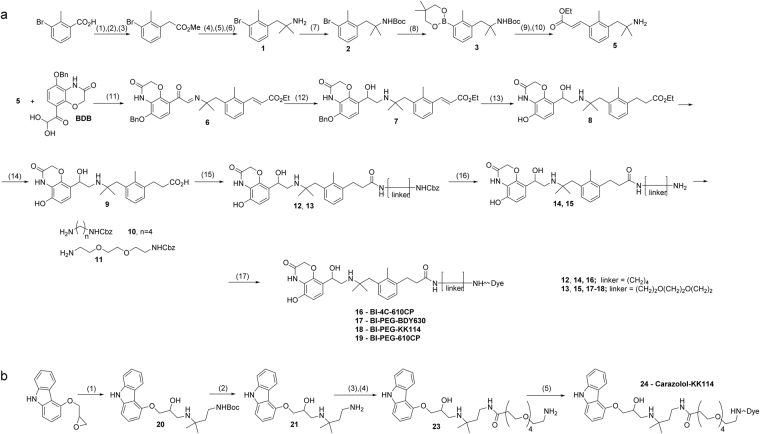
Synthesis of fluorescent ligands comprising BI-167107 (**a**) and carazolol (**b**) fragments. Reagents and conditions: (**a**) (1) SOCl_2_, reflux, 2 h; (2) CH_2_N_2_, Et_3_N, Et_2_O, 0 °C to rt, 3 h; (3) C_6_H_5_CO_2_Ag, Et_3_N, MeOH, ultrasound, 8 °C, 10 min; (4) MeMgBr, Et_2_O, 0 °C to rt, 3 h; (5) ClCH_2_CN, H_2_SO_4_, AcOH, 0 °C, 1 h; (6) thiourea, AcOH, EtOH, reflux, overnight; (7) Boc_2_O, NaHMDS, −20 °C to rt, THF, 1 h; (8) bis(neopentyl glycolato)diboron, Pd(dppf)Cl_2_, KOAc, dioxane, 110 °C, 4 h; (9) *trans*-BrCH = CHCO_2_Et, Pd(dba)_2_, SPhos, K_3_PO_4_, *n*-BuOH, 100 °C, 20 h; (10) TFA, DCM, 0 °C to rt, 16 h; (11) EtOH, 70 °C, 15 min; (12) NaBH_4_, EtOH/THF, 0 °C to rt, 1 h; (13) 10% Pd/C, H_2_, EtOH/MeOH, rt, overnight; (14) NaOH, EtOH/H_2_O, 0 °C to rt, 3 h; (15) H_2_N(CH_2_)_4_NHCbz or H_2_N-PEG_2_-(CH_2_)_2_NHCbz, PyBOP, DIEA, DMSO or DMF, rt, 1 h; (16) 10% Pd/C, H_2_, THF, rt, 24 h; (17) dye-NHS ester, Et_3_N, DMF, rt, 1 h. (**b**) (1) BocNH(CH_2_)_2_C(CH_3_)_2_NH_2_, EtOH, 90 °C, 4 h; (2) 4 M HCl in dioxane, EtOAc, rt, 1 h; (3) Boc-NH-PEG_4_-(CH_2_)_2_COOH, HATU, Et_3_N, DMSO, rt, 1 h; (4) TFA, DCM, rt, overnight; (5) dye-NHS ester, Et_3_N, DMF, rt, 1 h. PEG = -(CH_2_)_2_O-.

A common precursor of BI-167107 fluorescent derivatives was prepared by reductive amination of 5-(benzyloxy)-8-(dihydroxyacetyl)-2*H*-1,4-benzoxazin-3(*4*H)-one (BDB) with phenylethylamine **5** (compound **9**, Fig. 2a). BDB synthesis was performed as described by Bonnert *et al*.^18^ with the last step following the modified procedure by Floyd *et al*.^19^. The starting tertiary amine **1** was prepared in ten steps from commercially available 3-bromo-2-methylbenzoic acid via an Arndt-Eistert homologation to 2-(3-bromo-2-methylphenyl)acetate^20^, followed by a double Grignard addition to the ester and a Ritter reaction of the resulting tertiary alcohol^21^. Boronate **3** was prepared from *N*-Boc-protected amine **1** using Miyaura borylation, and Suzuki coupling of **3** with ethyl *trans*-3-bromoacrylate^22^ followed by deprotection afforded amine **5**. A common precursor of fluorescent derivatives of BI-167107 was prepared by reductive amination of BDB with phenylethylamine **5** (Fig. 2a).

The Schiff base **6** ^23^ obtained from BDB and amine **5** was selectively reduced with NaBH_4_. Removal of the benzyl protecting group and reduction of the double bond were performed simultaneously, and basic hydrolysis of ester **8** afforded the key BI-167107 analog **9** with a free carboxylic acid function. Attachment of linkers **10** and **11** followed by deprotection resulted in primary amines **14** and **15** ready for the dye conjugation (Fig. 2a).

As fluorescent tags, we have selected a lipophilic BODIPY 630/650-X^11^ dye, hydrophilic negatively charged rhodamine KK114^16^ and carbopyronine 610CP^17^, which is sensitive to the polarity of the environment. The conjugates of BODIPY dyes have been successfully applied in the studies of receptor-ligand complexes^11,24–27^, while both KK114 and 610CP are suitable for STED microscopy in fixed and living cells^17,28^. The red emission of the chosen fluorophores does not overlap with the cellular autofluorescence (<560 nm), permits simultaneous fluorescence detection of cyan-, green- and yellow-emitting fluorescent proteins and is suitable for terbium cryptate time-resolved fluorescence energy transfer (TR-FRET) assays (Supplementary Table 1, Supplementary Fig. 1).

The attachment of fluorescent dyes BODIPY 630/650-X, KK114 and 610CP to the BI-167107 derivatives resulted in a series of biologically active molecules **16**–**19** (Fig. 2a **;** see Fig. 1e for the final structures).

### Synthesis of Carazolol Derivatives

(*R*)-carazolol is known to be a less physiologically active enantiomer and (*S*)-carazolol has a lower β_2_/β_1_AR selectivity than (*R*)-enantiomer^29,30^. We performed the study with racemic carazolol derivatives to investigate whether they can be utilized as markers for βARs. The probes were prepared from the carazolol derivative **21** with a terminal primary amino group (Fig. 2b). Compound **21** was obtained from 4-(glycidyloxy)carbazole by nucleophilic oxirane ring opening with *tert*-butyl N-[3-amino-3-methylbutyl]carbamate^31^ followed by deprotection. Initially, a 610CP-carazolol probe was prepared by direct attachment of 610CP dye to the amino group of **21**, but was found to be unstable in aqueous buffers under ambient conditions. The polyethylene glycol (PEG_4_) linker was therefore introduced followed by the second deprotection and attachment of the fluorescent dye KK114 (see Fig. 1e for the final structure of **24**, carazolol-KK114).

### Characterization of fluorescent ligands, cAMP assay and *K*_*d*_^*app*^ determination

**Figure 3:**
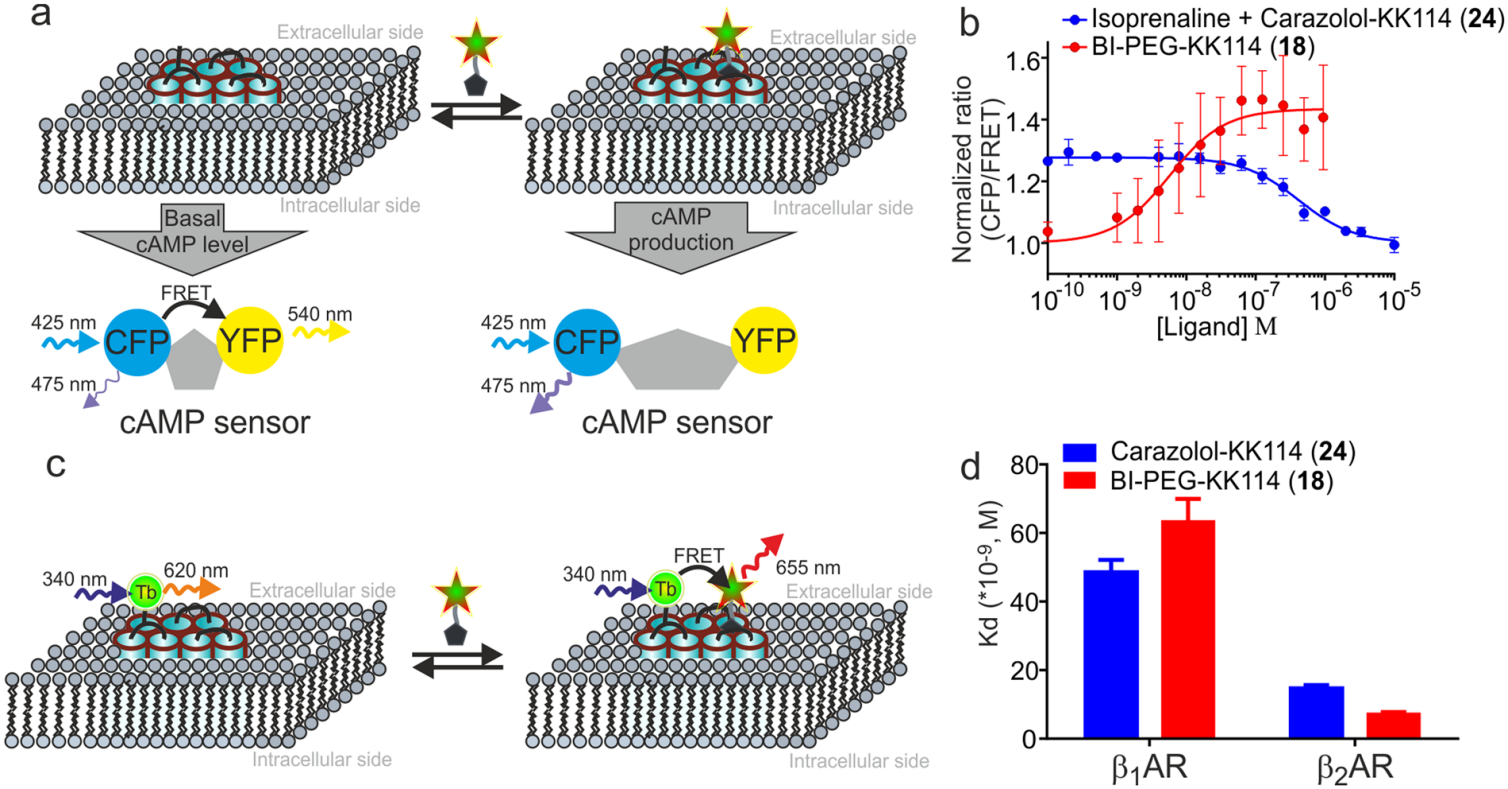
Determination of *K*_*d*_^*app*^ and cellular cAMP changes induced by βAR fluorescent ligands. (**a**) Schematic representation of the fluorescent cAMP biosensor ^T^Epac^VV^ mechanism of action. Basal cAMP level is present in the unstimulated cells resulting in a low mTurqouise (CFP) emission and large FRET signal. Addition of the agonist leads to production of cAMP, increase of CFP emission and decrease in FRET efficiency. (**b**) Intracellular cAMP sensor response; red line – the dose response curve of BI-PEG-KK114 (**18**) in HEK 293 expressing 3′,5′-cAMP FRET sensor cells. The cells were treated with increasing concentrations of BI-PEG-KK114 (**18**); blue line –displacement of isoprenaline with carazolol-KK114 (**24**) in HEK293 cells expressing biosensor ^T^Epac^VV^. The cells were treated with 17 nM isoprenaline and increasing concentrations of carazolol-KK114 (**24**). Sensor response *R*(*CFP/FRET*) was normalized to the *R*(*CFP/FRET*) of DMSO sample. All data correspond to 30 min incubation time; the estimated IC_50_ of carazolol-KK114 (**24**) from this experiment is 444 ± 79 nM. Data points are mean ± SD, n = 3 independent experiments. (**c**) Schematic representation of receptor-ligand saturation binding assay performed with Tag-lite (Cisbio) β_1_AR or β_2_AR Lumi4-Tb cryptate-labeled transfected cells with detection by Time-Resolved Fluorescence Energy Transfer (TR-FRET). (**d**) *K*_*d*_^*app*^ values of fluorescent ligands BI-PEG-KK114 (**18**) and carazolol-KK114 (**24**) obtained in TR-FRET assay. The cells were treated with fluorescent ligand and incubated for 2 h at room temperature before readout. Data points are mean ± SD, n = 4 independent experiments.

To evaluate the functional potency of the novel fluorescent ligands, we used HEK 293 expressing 3′,5′-cAMP FRET sensor (^T^Epac^VV^)^32^. In addition, these cells are known to endogenously express β_2_AR^33,34^. Upon cAMP binding, the sensor undergoes a conformational change resulting in a decrease of the FRET efficiency between two fluorescent proteins: mTurquoise - a version of cyan fluorescent protein (CFP) as the donor, and yellow fluorescent protein (YFP) as the acceptor (Fig. 3a). As a positive control, we used a nonselective β_1_/β_2_ adrenoceptor agonist isoprenaline. The registered CFP/FRET ratio decreased rapidly after the ligand addition, demonstrating agonist-promoted desensitization of the receptors^34–36^. Importantly, fitted half maximal effective concentration (EC_50_) did not significantly change during at least 60 min, thus allowing comparative studies of the ligands potencies (Supplementary Fig. 2a,b). We found that the EC_50_ of BI-167107 is one order of magnitude higher compared to isoprenaline (0.15 ± 0.02 nM vs 1.2 ± 0.4 nM) and its maximal cAMP sensor response is similar to isoprenaline after 30 min incubation time at room temperature (Supplementary Fig. 2c). Upon addition of fluorescent BI-167107 derivatives, we detected a concentration dependent stimulation of intracellular cAMP production, as indicated by a decrease in FRET efficiency of the sensor (Table 1, Fig. 3b, Supplementary Fig. 3). The EC_50_ values for all fluorescent ligands lie in nanomolar or subnanomolar range, demonstrating that the new probes retain sufficient affinity for the high quality staining of βARs even with bulky functional groups appended at the position 3 of BI-167107 (Fig. 1e, Table 1).

**Table 1:** Properties of βAR agonists and antagonists and imaging performance of fluorescent ligands.

Name	EC_50_ (mean ± SD, nM)		Staining performance	Receptor internalization	Competitive displacement
**Agonists**					
Isoprenaline	1.2 ± 0.4		—	Yes	—
BI-167107	0.15 ± 0.02		—	Yes	—
BI-4C-610CP (**16**)	7.3 ± 1.4		Medium	Yes	N.d.
BI-PEG-BDY630 (**17**)	0.18 ± 0.03		Strong	Yes	Background
BI-PEG-KK114 (**18**)	5.7 ± 2.2		Strong	Yes	Yes
BI-PEG-610CP (**19**)	0.62 ± 0.09		None	N.d.	N.d.
**Antagonists**					
**Name**	**IC**_**50**_ **(mean ± SD, nM)**	***K***_***i***_^***app***^ **(mean ± SD, nM)**	**Staining performance**	**Receptor internalization**	**Competitive displacement**
Carazolol	2.6 ± 0.7	0.17 ± 0.10	—	—	—
ab118171	74.7 ± 36.3	4.8 ± 3.6	Strong	Weak	Background
Carazolol-KK114 (**24**)	2451 ± 494	161.6 ± 76.0	Strong	Weak	Yes

We compared the efficiencies of carazolol-KK114 (**24**) and of commercial (*S*)-carazolol fluorescent derivative ab118171 (Abcam plc) as cAMP production inhibitors in HEK293 cells expressing the fluorescent biosensor ^T^Epac^VV^ (Supplementary Fig. 4). ab118171 is a red-emitting (λ_abs_ = 633 nm, λ_em_ = 650 nm) β_1_AR and β_2_AR antagonist bearing a BODIPY 630/650 fluorophore (Fig. 1e). Both fluorescent probes were able to reduce cAMP concentration in isoprenaline-stimulated cells as judged from decrease of ^T^Epac^VV^ sensor CFP/FRET ratio (Supplementary Fig. 4). The apparent competitive inhibitory constants (*K*_*i*_^*app*^) were 4.8 ± 3.6 nM for ab118171 and 161.6 ± 76.0 nM for carazolol-KK114 (**24**), as calculated from the fitted half maximal inhibitory concentrations (IC_50_) right after addition of ligands using the Cheng-Prusoff equation^37^ (Table 1). If the measurements were performed 30 min after addition of the ligands, the *K*_*i*_^*app*^ of carazolol-KK114 (**24**) decreased to 29.3 ± 15.0 nM (Fig. 3b). This change might be due to the complex processes involving receptor desensitization, internalization and/or activation of cAMP phosphodiesterases^34^, and similar phenomena were observed earlier^38^.

To confirm whether the affinity of carazolol-KK114 (**24**) is sufficient for the intended imaging experiments and to evaluate its β_2_/β_1_AR selectivity, we performed a TR-FRET assay in Tag-lite β_1_AR or β_2_AR transfected cells (Cisbio) labeled with Tb^3+^ cryptate (Lumi4-Tb) (Fig. 3c)^39^. Carazolol-KK114 (**24**) demonstrated high affinity for β_2_AR with the determined apparent dissociation constant (*K*_*d*_^*app*^) of 14.7 ± 0.9 nM, and showed approximately 3-fold binding preference to β_2_AR over β_1_AR (Fig. 3d, Supplementary Fig. 5a). In comparison with that, the BI-167107 derivative bearing a KK114 fluorophore (**18**) demonstrated almost 9-fold binding selectivity in the TR-FRET assay with *K*_*d*_^*app*^ equal to 63.4 ± 6.6 nM for β_1_AR versus 7.2 ± 0.6 nM for β_2_AR. (Fig. 3d, Supplementary Fig. 5b). The high affinities of both fluorescent ligands enabled the imaging experiments.

### Fluorescence microscopy on β_2_AR-YFP expressing U2OS cells

Intending to apply our fluorescent ligands for the visualization of the ligand-receptor interactions using confocal and STED microscopy, we examined binding specificity of compounds **16**–**19** and **24**, for β_2_AR sites in U2OS cells overexpressing a β_2_AR-YFP fusion protein. We found that BI-PEG-610CP (**19**) produced almost no signal despite its low EC_50_ (Table 1, Supplementary Fig. 6a). The membrane-permeant dye 610CP is sensitive to the polarity of the medium and is known to undergo spirolactonization in hydrophobic environments^17^ with a loss of fluorescence intensity. We assumed that the fluorophore of BI-PEG-610CP (**19**) is embedded into the lipophilic environment of the plasma membrane upon binding to the receptor. Indeed, the probe BI-4C-610CP (**16**), where 610CP dye is attached via a shorter aliphatic linker, provided satisfactory images upon binding to the receptors (Supplementary Fig. 6b).

**Figure 4:**
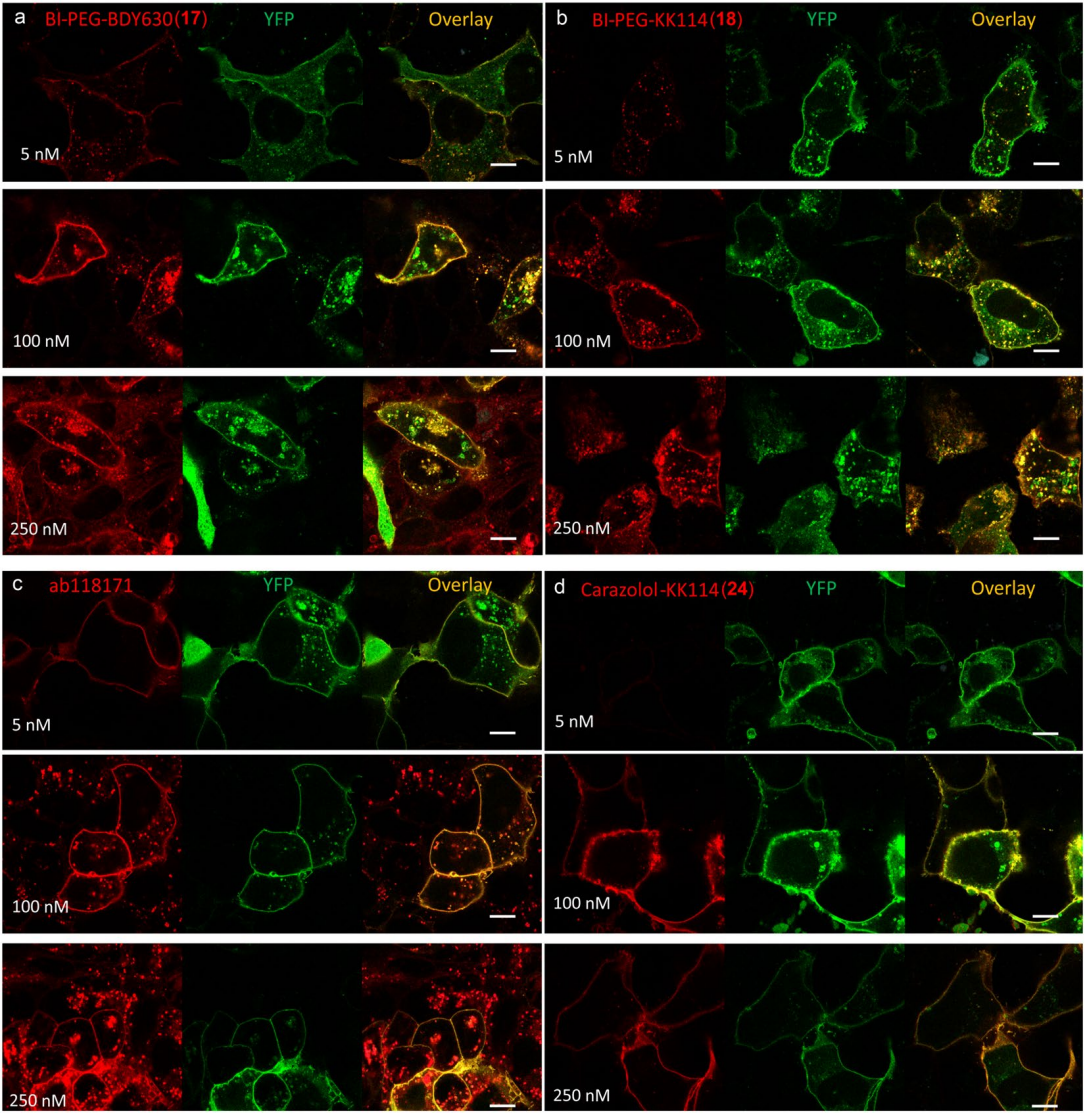
Confocal images of living U2OS cells expressing the β_2_AR-YFP fusion protein stained with fluorescent ligands. Cells were incubated for 40 min at room temperature in the presence of 5 nM, 100 nM or 250 nM of either: (**a**) BI-PEG-BDY630 (**17**), (**b**) BI-PEG-KK114 (**18**) (**c**) ab118171 or (**d**) carazolol-KK114 (**24**) in growth medium and washed two times before imaging. Images were obtained on a Leica SP8 microscope using identical settings for each fluorescent ligand as described in the Methods section. The individual color channels and their overlay images are shown. Scale bars 10 µm.

After incubation of U2OS cells with the ligands BI-PEG-BDY630 (**17**) and BI-PEG-KK114 (**18**) at concentration range of 5–250 nM for 30–40 min at 25 °C, bright labeling was observed in confocal images, overlapping specifically with the β_2_AR-YFP fluorescence signal (Fig. 4a,b). We also detected punctate staining inside the cells resulting from the agonist-induced β_2_AR internalization^40^. 100 nM BI-PEG-BDY630 (**17**) labelled the cells efficiently, whereas at higher concentration (250 nM) unspecific fluorescence was detected in the areas outside the β_2_AR-YFP expressing cells (Fig. 4a). The hydrophilic ligand BI-PEG-KK114 (**18**) performed well at both concentrations of 100 nM and 250 nM (Fig. 4b). 5 nM of ab118171 showed specific membrane localized fluorescence, but the images obtained at higher 100 nM and 250 nM concentrations demonstrated non-specific dotted background inside as well as outside the cells (Fig. 4c). In contrast, carazolol**-**KK114 (**24**) showed specific staining at all tested concentrations (Fig. 4d).

### Competitive binding and comparison with a commercial βAR fluorescent ligand

**Figure 5:**
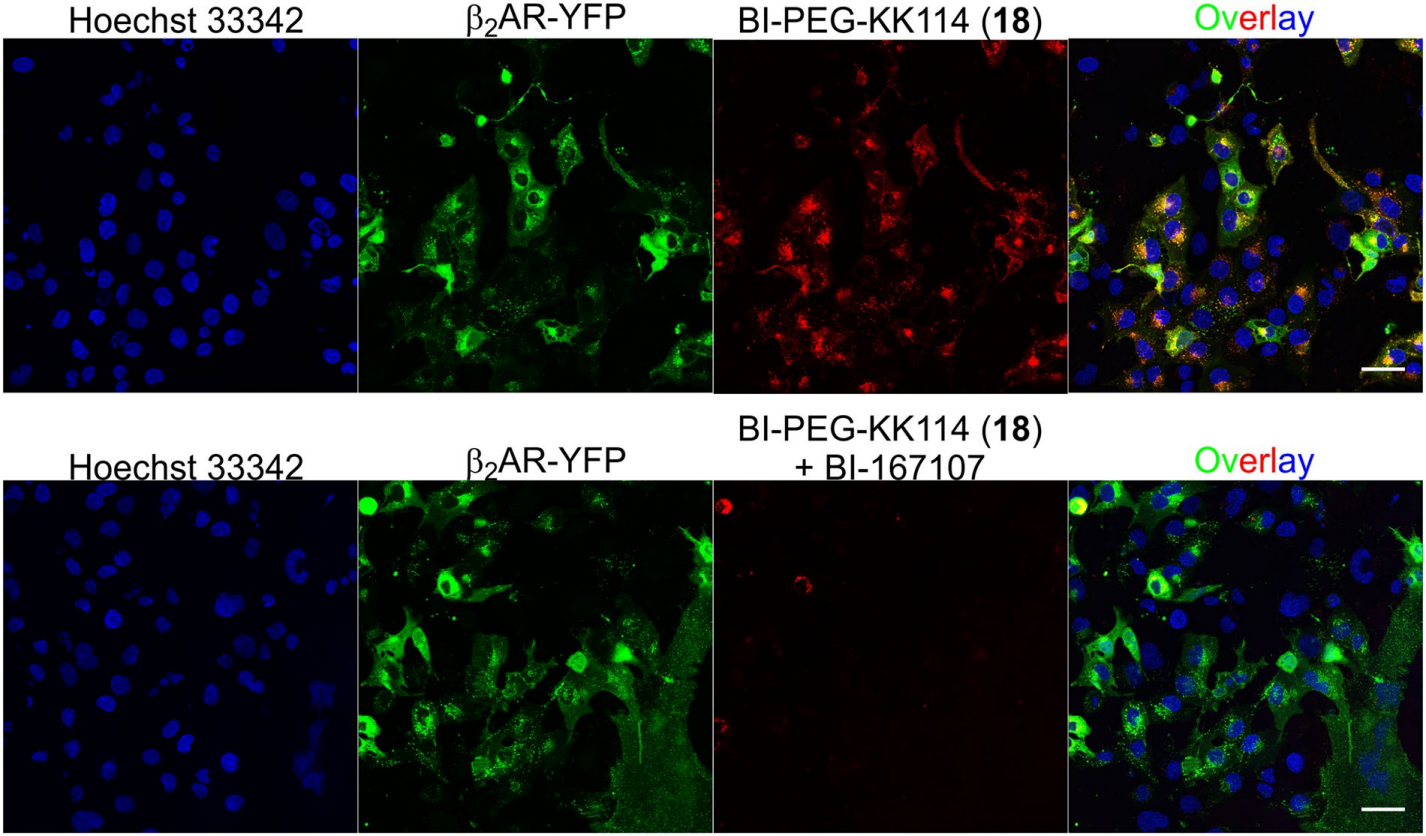
Competitive displacement of BI-PEG-KK114 (**18**) βAR fluorescent probe with non-fluorescent BI-167107 ligand. Living U2OS cells expressing β_2_AR-YFP fusion protein were incubated for 30 min with a mixture of 100 nM BI-PEG-KK114 (**18**) and 10 µM BI-167107 in growth medium in presence of 0.1 µg/ml Hoechst 33342 and washed two times before imaging on a Leica SP8 microscope. The individual color channels and their overlay images are shown. Scale bars 50 µm.

To investigate the specificity of our βAR probes, we performed a competitive binding experiment. In the presence of large excess of the unlabeled ligand together with the fluorescent probe, the former is predominantly bound to the receptors and no specific fluorescence signal is observed. We performed the competitive binding experiments in U2OS cells expressing β_2_AR-YFP with ≥ 100-fold excess (10 µM) of unlabeled BI-167107 or carazolol over the corresponding fluorescent ligands. Low background fluorescence was observed for 100 nM of BI-PEG-BDY630 (**17**) and no signal was registered for 100 nM BI-PEG-KK114 (**18**) in the presence of excess BI-167107. These observations suggested their specificity for β_2_AR (Fig. 5, Supplementary Fig. 7). On the contrary, when 5 nM solution of ab118171 probe together with 2000-fold excess of carazolol (10 µM) was applied, U2OS β_2_AR-YFP cells still demonstrated fluorescence signal (Supplementary Fig. 8). Application of the same probe at the manufacturer’s recommended concentration (100 nM) resulted in additional punctate staining inside and outside β_2_AR-YFP expressing U2OS cells, suggesting even larger off-target labeling (250 nM; Fig. 4). Carazalol-KK114 (**24**) performed much better, showed no background fluorescence in the competitive displacement experiment (Supplementary Fig. 8). The EC_50_ and IC_50_ values of βAR agonists and antagonists, respectively, and the performance of the fluorescent ligands in imaging are summarized in Table 1.

### Inter- and intramolecular interactions in probe solutions

**Figure 6:**
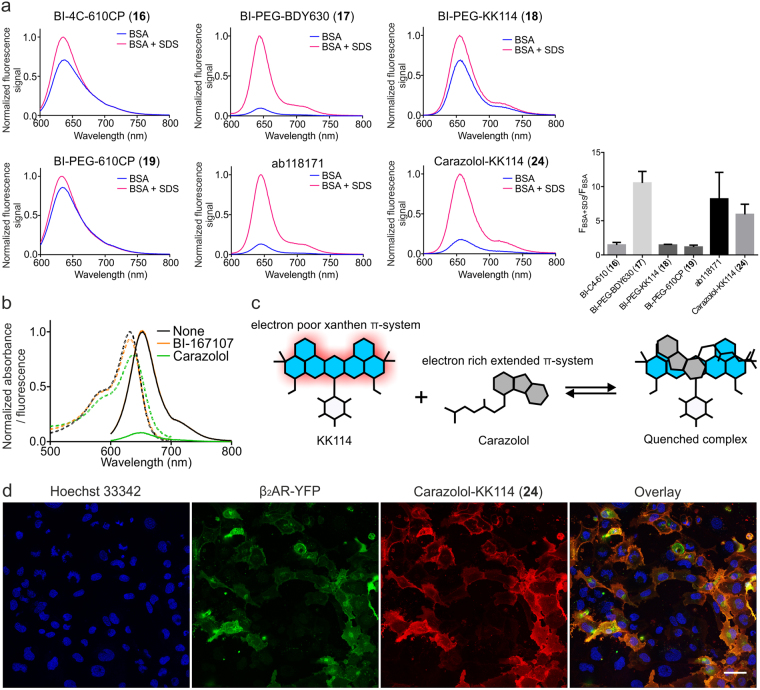
Fluorogenic behavior of the βAR fluorescent probes. (**a**) Fluorescence increase upon addition of sodium dodecyl sulfate (SDS). SDS was added to solutions of the probes in PBS containing 1 mg/ml BSA to 0.1% final concentration. The spectra are averaged from three independent experiments and normalized to F_BSA+SDS_ at fluorescence emission maximum. The bars represent the calculated ratios F_BSA+SDS_/F_BSA_ of fluorescence emission intensities at 635 nm for 610CP, 645 nm for BODIPY 630/650-X and 655 nm for KK114 conjugates. Fluorescence increase is presented as a mean ± SD, n ≥ 3 independent experiments. (**b**) Absorption and emission spectra of KK114-COOH in PBS (pH 7.4) recorded before and after addition of 1000-fold molar excess of either carazolol or BI-167107 (average of 3 independent experiments). (**c**) Proposed mechanism of fluorescence quenching caused by carazolol. Intramolecular quenching may be induced by π-stacking between the carazolol and KK114 aromatic systems. (**d**) No-wash imaging experiment. Living U2OS cells expressing β_2_AR-YFP fusion protein were incubated for 90 min in presence of 100 nM carazolol-KK114 (**24**) in growth medium. Confocal images were recorded on a Leica SP8 microscope without washing off the excess of probe. Scale bar 50 µm.

We further investigated non-specific staining by BODIPY 630/650-containing ligands ab118171 and BI-PEG-BDY630 (**17**), which may be due to self-aggregation and/or nonspecific binding to biomolecules. The self-aggregation of fluorescent probes can be evaluated by fluorescence recovery in PBS solutions upon addition of an anionic surfactant, such as sodium dodecyl sulfate (SDS)^41–43^. Indeed, we have observed that addition of SDS to solutions of ab118171 increased the fluorescence emission intensity and resulted in a red shift of the absorption and fluorescence maxima (Fig. 6a, Supplementary Table 2).

Moreover, a 10 nm blue shift for both the absorption and emission maxima was observed upon addition of BSA to the PBS solution of non-conjugated BODIPY 630/650 dye (Supplementary Fig. 9). This behavior indicates that the lipophilic BODIPY fluorophore can bind nonspecifically to cellular proteins.

Surprisingly, we also detected fluorescence intensity increase in the solution of carazolol-KK114 (**24**) upon addition of SDS. The fluorescence quantum yield (QY) of carazolol-KK114 (**24**) probe (0.16) measured in PBS (pH 7.4) is significantly lower than that of the free dye (0.50) (Supplementary Table 3). Both findings indicate that intramolecular quenching might occur via electron transfer from the carbazole residue (donor) to KK114 fluorophore (acceptor) in the excited state. The same phenomenon was observed intermolecularly upon mixing the free KK114 dye (1 µM) with excess of carazolol (1 mM) in PBS solution (Fig. 6b, Supplementary Table 3). In contrast, almost no quenching was observed for BI-167107 derivatives, except for BI-PEG-BDY630 (**17**), as evidenced by little to no emission change upon addition of SDS to the solution of BI-PEG-KK114 (**18**) (Fig. 6a). Accordingly, addition of 1000-fold excess of BI-167107 to the free KK114 dye solution in PBS did not significantly change the position of the emission maximum and QY of the dye (Fig. 6b, Supplementary Table 3).

### Fluorogenicity of carazolol-derived fluorescent probes allows no-wash imaging experiments

Fluorogenic behavior (fluorescence enhancement upon binding of a probe to its target) is a desirable property of fluorescent probes, which simplifies imaging experiments by increasing the signal to noise ratio and, in some cases, allows the omitting of the washing steps. Expecting that the intramolecular quenching of carazolol-KK114 (**24**) fluorescence would result in its fluorogenic behavior, we attempted a no-wash imaging of β_2_AR-YFP expressing U2OS cells and compared the performance of KK114- and BODIPY 630/650-carazolol probes. The specific staining was observed after the cells were incubated with 100 nM solution of carazolol-KK114 (**24**) (Fig. 6d). In contrast, ab118171 showed strong off-target staining under no-wash conditions at much lower concentrations (5 nM; Supplementary Fig. 10).

### pH dependence of the βAR ligands’ fluorescence

Binding of the βAR ligands to their receptors is generally followed by internalization of the receptors via endocytosis. The internalized fluorescent ligand may be exposed to considerably lower pH. To evaluate the pH sensitivity, we recorded the fluorescence spectra of various βAR ligands within the physiologically relevant pH range 4.7–8.0 (Supplementary Fig. 11). The fluorescence of carazolol-KK114 (**24**) and BI-PEG-KK114 (**18**) did not change significantly with varying pH. At pH ~7, corresponding to cytoplasmic environment, the fluorescence of βAR antagonist carazolol-KK114 (**24**) was slightly lower than at pH ~5, which is characteristic to endosomal or lysosomal lumen^44^, maintaining about 90% of its maximal fluorescence intensity. In contrast, βAR agonist BI-PEG-KK114 (**18**) showed stable fluorescence intensity over pH interval 4.7–6.0, but at pH 8.0 its emission intensity decreased by 30%. BI-PEG-BDY630 (**17**) ligand was found to be more pH-sensitive with highest fluorescence intensities around pH 6.0–7.0 and about 40% fluorescence decrease at pH 4.7 and 8.0. ab118171 was the most pH-sensitive ligand with almost two-fold fluorescence reduction at pH 7.0 compared to that at acidic pH 4.7.

### STED nanoscopy of U2OS cells overexpressing β_2_ARs

**Figure 7:**
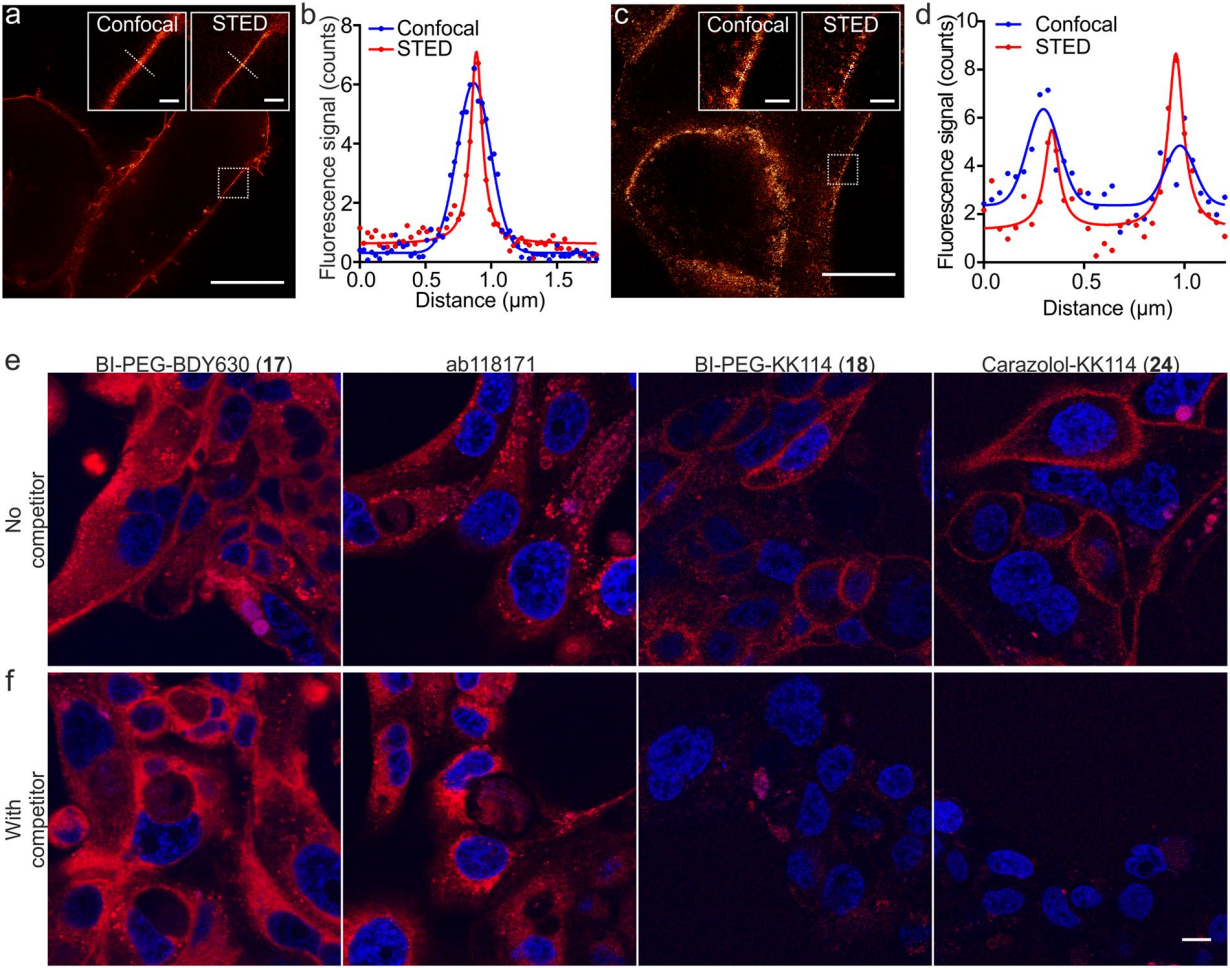
Images of living cells expressing β_2_AR stained with fluorescent ligands. (**a**) STED image of a living U2OS cell expressing β_2_AR-YFP fusion protein labeled with carazolol-KK114 (**24**). The cells were incubated for 40 min in growth medium in presence of 100 nM carazolol-KK114 (**24**) and washed two times before imaging on an Abberior STED 775 QUAD scanning microscope. The inserts show zoomed-in confocal and STED images of the selected fragment (dotted square). Scale bars: 10 µm for the large image, 1 µm for the inserts. (**b**) Line profiles taken along the dotted line across the cell membrane in the zoomed-in regions. The confocal profile is fitted to Gaussian distribution, STED profile is fitted to Lorentz distribution. (**c**) STED image of live CAPAN-1 cells stained with 100 nM BI-PEG-KK114 (**18**). The cells were incubated for 40 min in growth medium in presence of a fluorescent ligand and washed two times before imaging on an Abberior STED 775 QUAD scanning microscope. The inserts show zoomed-in confocal and STED images of the selected fragment (dotted square). Scale bars: 10 µm for the large image, 1 µm for the inserts. (**d**) Line profiles taken along the dotted line across the cell membrane in the zoomed-in regions. The profile is fitted to multiple Gaussian distribution. (**e**,**f**) Competitive binding experiment with excess of non-fluorescent ligands: (**e**) confocal images of live CAPAN-1 cells stained with fluorescent ligands: ab118171 (5 nM), BI-PEG-BDY630 (**17**) (100 nM), carazolol-KK114 (**24**) (100 nM), BI-PEG-KK114 (**18**) (100 nM). (**f**) Same as (**e**), in the presence of excess (10 µM) of non-fluorescent ligands: carazolol for antagonists ab118171 and carazolol-KK114 (**24**); BI-167107 for agonists BI-PEG-BDY630 (**17**) and BI-PEG-KK114 (**18**). Scale bars 10 µm.

With the characterization of the probes accomplished, we studieded the applicability of the fluorescent probes in STED nanoscopy. The images of U2OS cells expressing β_2_AR-YFP were acquired after 30–40 min incubation with 5 nM of ab118171 or with 100 nM of BI-PEG-BDY630 (**17**), BI-PEG-KK114 (**18**) and carazolol-KK114 (**24**) (Fig. 7a; Supplementary Fig. 12a). We observed significant improvement of optical resolution for all fluorescent probes. Cell membrane cross-sections demonstrated similar apparent thicknesses of ~100 nm for the side view of the receptor layer, as compared to ~270 nm layer thickness observed with confocal imaging (Fig. 7a,b; Supplementary Fig. 12b). We expected the same resolution improvement in lateral direction, but the large overexpression of β_2_AR-YFP construct resulting in high β_2_AR density made this measurement impossible, necessitating tests in the cell lines with lower endogenous expression of β_2_AR.

Carazolol is reported to act as a βAR antagonist and inhibits cAMP production as well as receptor internaziation^45^. In contrast, BI-167107 as an agonist promotes cAMP production and triggers receptor internalization^12,13^. Our fluorescent probes retained these antipodal properties and allowed modulation of cellular response in the course of imaging experiments. For example, addition of carazolol-KK114 (**24**) to the cells did not change intracellular cAMP level and internalization of receptors. On the contrary, addition of BI-PEG-KK114 (**18**) triggered extensive βAR internalization (Supplementary Figure 13).

### Fluorescent probes stain selectively at the endogenous β_2_AR expression level

To study the selectivity of β_2_AR staining at the endogenous expression levels^46^, which are much lower compared to those in transiently β_2_AR expressing U2OS cells, we tested the performance of BI-167107 probes in pancreatic CAPAN-1 cells (Fig. 7c–f). In competitive binding experiments with unlabeled BI-167107 (10 µM, 100-fold), strong off-target staining for BI-PEG-BDY630 (**17**) and no background fluorescence for BI-PEG-KK114 (**18**) were observed, confirming superior staining specificity for the KK114 probe **18** at the same concentration (100 nM) (Fig. 7e,f; Supplementary Fig. 14). It was not possible to achieve full displacement of 5 nM ab118171 probe with 10 µM carazolol (2000-fold excess). In contrast, 100 nM carazolol-KK114 (**24**) was efficiently displaced by 10 µM carazolol (100-fold excess), demonstrating highly specific binding at the receptor sites (Fig. 7e,f). We observed plasma membrane localized receptor staining in CAPAN-1 cells after incubation with 100 nM solutions of BI-PEG-KK114 (**18**) or carazolol-KK114 (**24**) ligands. Low density of β_2_AR in the plasma membrane of CAPAN-1 cells allowed us to measure of the lateral resolution in the STED images. When the cells were incubated with 100 nM BI-PEG-KK114 (**18**) ligand, we observed two-fold improvement in the resolution resulting in apparent structures of ~100 nm (Fig. 7d; Supplementary Table 4).

## Discussion

We have designed, synthesized and characterized five red-emitting fluorescent ligands acting as functional agonists and antagonists of the human βAR receptors. Our probes are based on the agonist BI-167107 and antagonist carazolol as racemates, conjugated to BODIPY 630/650-X, KK114 and 610CP fluorophores (λ_em_ > 600 nm). We discovered that the use of 610CP-conjugated βAR ligands as fluorescent probes is limited due to the environment sensitivity of this dye and its ability to form non-fluorescent spirolactone in hydrophobic media. While such environment-based fluorogenicity is advantageous for labeling nuclear^47^ and cytoplasmic proteins^17^, the 610CP dye seems poorly suitable for targets located within the plasma membrane. Despite the fact that the 610CP derivatives of BI-167107 (**16** and **19**) were functionally active and possessed low nanomolar EC_50_, the staining of β_2_AR-YFP expressing U2OS cells was only possible at micromolar concentrations, or not possible at all.

BI-PEG-BDY630 (**17**) and commercial ab118171 probes, containing a lipophilic BODIPY 630/650 fluorophore (Fig. 1e) found to be suitable for STED nanoscopy, showed high affinity (Table 1). However, neither ligand can be recommended for labeling of βARs at low expression levels because of the marked off-target staining. The complex behavior of these probes may be due to several associated factors: intramolecular quenching, aggregation and/or nonspecific binding of the relatively hydrophobic BODIPY 630/650 fluorophore (Fig. 6a).

The βAR fluorescent ligands containing the hydrophilic dye KK114 demonstrated much better performance in cellular environment. Among the four BI-167107 probes, the KK114-derived fluorescent agonist **18** provided the highest-contrast and the brightest images in living cells at nanomolar concentrations. Remarkably, its binding affinity and functional activity were comparable to that of the parent ligand (Fig. 3). Similarly, the antagonist carazolol-KK114 (**24**) provided excellent fluorescence images at nanomolar concentrations, even though the functional potency of probe **24** was inferior to that of the commercial ab118171 (Supplementary Fig. 4). Addition of SDS to the solution of carazolol-KK114 (**24**) increased the emission intensity, due to presumed disruption of intramolecular quenching caused by π-stacking of carazolol and KK114 aromatic systems. This behavior allowed no-wash imaging experiments in cells overexpressing the target protein (Fig. 6d).

The combination of BI-167107 and carazolol-based probes allowed testing of the functional behavior of βAR. We observed that BI-167107, but not the carazolol derivatives, trigger extensive internalization of βAR receptors in U2OS cells expressing β_2_AR-YFP, thus confirming that U2OS cells are able to respond correctly to GPCR stimulation.

We have demonstrated the applicability of the new fluorescent probes in live-cell STED nanoscopy. The observed up to three-fold resolution improvement allows detection of receptor distributions in the plasma membrane of living U2OS cells expressing β_2_AR-YFP (Supplementary Table 4). Importantly, the introduced KK114-containing βAR fluorescent probes (**18** and **24**) provide sufficient brightness and high staining specificity at the native β_2_AR expression levels in CAPAN-1 cells (Fig. 7e,f). These probes also demonstrate moderate β_2_AR selectivity over β_1_AR, nine-fold for the agonist probe **18**, three-fold for the fluorescent antagonist **24** (Supplementary Fig. 5).

We expect that the newly developed probes will prove useful for direct observation and evaluation of physiological and pathological functions of βAR in a variety of βAR-expressing cells and tissues.

## Methods

### General materials and methods

Flash chromatography was performed on Merck Silica 60 0.04–0.063 mm (Cat. No. 815360) or using Biotage Isolera flash purification system with a cartridge and solvent gradient indicated. Reverse-phase chromatography was performed on Polygoprep 60–50 C18 (Macheray-Nagel, Düren, Germany; Cat. No. 711500). All chemicals were purchased from Sigma-Aldrich (Munich, Germany), Merck (Darmstadt, Germany), Alfa Aesar (Karlsruhe, Germany), J & W PharmLab LLC (Levittown, PA, USA) or TCI (Eschborn, Germany) and used as received, unless otherwise indicated. BODIPY 630/650-X succinimidyl ester (BDY630-X, SE) was purchased from Tocris (Bristol, UK). BODIPY 630/650 carboxylic acid (BDP-630/650 carboxylic acid) was purchased from Lumiprobe (Hannover, Germany). NMR spectra were recorded at ambient temperature with Agilent 400-MR spectrometer (MPI BPC Göttingen) at 400.06 MHz (^1^H) and 100.60 MHz (^13^C) and are reported in ppm. All ^1^H spectra are referenced to tetramethylsilane (*δ = *0 ppm) using the signals of the residual protons of CHCl_3_ (7.26 ppm) in CDCl_3_, acetone-*d*_5_ (2.05 ppm) in acetone-*d*_6_, CHD_2_OD (3.31 ppm) in CD_3_OD or DMSO-*d*_5_ (2.50 ppm) in DMSO-*d*_6_. ^13^C spectra are referenced to tetramethylsilane (*δ = *0 ppm) using the signals of the solvent: CDCl_3_ (77.16 ppm), acetone-*d*_6_ (CD_3_, 29.84 ppm), CD_3_OD (49.00 ppm) or DMSO-*d*_6_ (39.52 ppm). Multiplicities of signals are described as follows: s = singlet, d = doublet, t = triplet, q = quartet, m = multiplet or overlap of signals; br. s = broad signal. Coupling constants (*J*) are given in Hz. Low resolution mass spectra (50–3500 m/z) with electrospray ionization (ESI) were recorded on a Varian 500-MS spectrometer (Agilent) at MPI BPC Göttingen. High resolution mass spectra (ESI-HRMS) were recorded on a MICROTOF spectrometer (Bruker) equipped with ESI ion source (Apollo) and direct injector with LC autosampler Agilent RR 1200 at the Georg-August-Universität Göttingen. Liquid chromatography (HPLC) was performed using Knauer Smartline liquid chromatography system: two pumps (1000), UV-detector 2500, column thermostat 4000, mixing chamber and injection valve with 20 μL and 100 μL loops for analytical and preparative columns, respectively; 6-port-3-channel switching valve. Analytical column: Eurospher 100 C18, 5 μm, 250 × 4 mm; preparative column: Eurospher 100 C18, 5 μm, 250 × 16 mm; solvent A: acetonitrile + 0.1% v/v TFA, solvent B: H_2_O + 0.1% v/v TFA; temperature 25 °C. Analytical TLC was performed on ready-to-use silica gel 60 (F_254_) aluminium plates (Merck). Preparative TLC was performed on precoated thin-layer plates with silica gel for high performance TLC (HPTLC Silica gel 60 F_254_ 10 × 10 cm, with concentrating zone 10 × 2.5 cm;Merck, Darmstadt, Germany; Cat. No. 113727). Synthetic procedures and characterization of all compounds are given in the Supplementary Information.

Fluorescence quantum yields (absolute values) were obtained on a Quantaurus-QY absolute PL quantum yield spectrometer (model C11347-12, Hamamatsu) at ambient temperature (25 °C), excitation wavelength 580 nm; all measurements were performed in triplicates.

### Fluorescence and absorbance spectra

Fluorescence spectra of 1 µM solution of fluorescent dyes and ligands in PBS pH 7.4, PBS pH 7.4 + 1 mg/ml BSA (Sigma) and with or without 0.1% SDS (Acros Organics) were recorded on a multiwell plate reader Spark 20 M (Tecan) in half-area black 96-well plates (Greiner Bio-One, cat. 675076) at room temperature (25 °C) after 2 h incubation. Fluorescence emission was recorded from 600 nm to 800 nm with excitation at 580 nm. The emission bandwidth for all measurements was set to 5 nm. The spectra were averaged from 3 individual experiments. Ratios F_(BSA+SDS)_/F_(BSA)_ of fluorescence signals at 635 nm for 610CP, 645 nm for BODIPY 630/650-X and 655 nm for KK114 conjugates were calculated. The obtained results are summarized (Table 1 and Supplementary Fig. 7).

Absorbance spectra of 1 µM solutions of BODIPY 630/650 carboxylic acid (Lumiprobe, Cat. No. 45490) and KK114 carboxylic acid were recorded on a multiwell plate reader Spark 20 M (Tecan) in 96-well glass bottom plates (MatTek Corporation; Cat. No. PBK96G-1.5-5-F) at room temperature (25 °C) after 2 h incubation time. The spectra were averaged from 3 individual experiments and integrated from 500 to 750 nm.

### Fluorescence measurements of βAR ligands at different pH

pH dependence of βAR ligands fluorescence were recorded on a multiwell plate reader Spark 20 M (Tecan) in 96-well glass bottom plates (MatTek Corporation; Cat. No. PBK96G-1.5-5-F) in PBS solutions (pH 4.7–8.0) at room temperature (25 °C). The 100x stock solutions of ligands were prepared in DMSO.

### Cell culture

U2OS cells (ATCC) were cultured in high-glucose DMEM (Dulbecco’s Modified Eagle medium, Thermo Fisher Scientific) supplemented with GlutaMAX-1 (Thermo Fisher Scientific) and 10% fetal bovine serum (FBS) (Thermo Fisher Scientific) in a humidified 5% CO_2_ incubator at 37 °C. CAPAN-1 cells (ATCC) were cultured in McCoy’s 5a Medium Modified supplemented with 10% fetal bovine serum (FBS) (Thermo Fisher Scientific) in a humidified 5% CO_2_ atmosphere at 37 °C. Cells were split every 3–5 days or at confluence. Cells were seeded in glass bottom 12-well plates (MatTek Corporation). To express β_2_AR-YFP, U2OS cells were transiently transfected with plasmid pEYFP-β_2_AR (Addgene; Cat. No. 67146)^48^ using Lipofectamine 2000 according to reverse transfection protocol adapted for plasmid DNA^49^. We used 200,000 cells per well, 3 µg plasmid DNA and 3 µl of Lipofectamine 2000.

### cAMP studies

The intracellular cyclic adenosine monophosphate (cAMP) levels were determined with HEK 293 cells expressing a 3′,5′-cAMP FRET sensor. The cells were a generous gift from Ruud Hovius from École poly-technique fédérale de Lausanne (EPFL). The cAMP production induces a conformational switch in ^T^Epac^VV^ and causes a decrease in FRET efficiency, i.e. a decreased energy transfer between the two fluorescent proteins (donor mTurquoise and acceptor YFP)^32^. The FRET sensor is a cytoplasmic protein construct and responds to cAMP changes in the physiological range (0.1–100 mM).

HEK 293 cells expressing a 3′,5′-cAMP FRET sensor were grown in DMEM media supplemented with antibiotic G-418 (150–300 µg/ml) at 37 °C and in humidified atmosphere with 5% CO_2_. The culture was maintained by subculturing the cells every 4 to 5 days using the standard trypsinization procedure. Before measurements, cells were trypsinated and transferred in a 96-well glass bottom plate (MatTek Corporation; Cat. No. P96GC-1.5–5-F) to contain 10,000–30,000 cells per well in 200 µL of DMEM media. After 24 h incubation in 5% CO_2_ at 37 °C, the cells were washed with HBSS pH 7.2 (200 µL), and the wells were filled with 200 µL HBSS. The DMSO stock solutions of the fluorescent probes were diluted 5,000 times in HBSS and used in experiments. In the antagonist inhibition experiment, we used β_1_ and β_2_ adrenoceptor agonist isoprenaline (abcr GmbH) as a positive control causing cAMP elevation. Freshly diluted solution of isoprenaline with constant concentration (17 nM) in HBSS was used. The fluorescent ligand solutions of different concentrations in HBSS were premixed with the isoprenaline solution. 50 µL of a final solution was added to each well and the measurement was started upon addition and continued for 1 h in 10 min intervals between the readouts (inclusive).

The ligands were added using an automated pipette to initiate the sensor response. FRET readout from the cAMP sensor was performed on a multiwell plate reader Spark 20 M (Tecan) at room temperature (25 °C). Fluorescence readout amplitudes were calculated from time series for different ligand concentrations, following background subtraction (media only). The donor/acceptor emission ratios were calculated for each ligand concentration (donor: exc. 425 ± 15 nm, em. 475 ± 130 nm; acceptor: exc. 425 ± 15 nm, em. 540 ± 130 nm), including normalization with the donor/acceptor ratio from wells without cells. Data were derived from 3 independent experiments and each independent experiment was performed in triplicate.

Sensor response *R*(*CFP/FRET*) was calculated using (1):

1 $$R(\frac{CFP}{FRET})=({F}_{i}^{{\rm{mTurquoise}}}-{F}_{bkg}^{{\rm{mTurquoise}}})/({F}_{i}^{FRET}-{F}_{bkg}^{FRET})$$

where *F*_*i*_^mTurquoise^ – mTurquoise fluorescence intensity in the wells with cells; *F*_*bkg*_^mTurquoise^ - mTurquoise fluorescence intensity in the control wells without cells; *F*_*i*_^*FRET*^ – FRET signal intensity in the wells with cells; *F*_*bkg*_^*FRET*^ – FRET signal intensity in the control wells without cells. For every result, *R*(*CFP/FRET*) ratio was normalized to the *R*(*CFP/FRET*) response of DMSO sample.

EC_50_ values were determined from the dose-response curves obtained after 30 min incubation of cells in the presence of agonist; unless otherwise stated, IC_50_ values were determined from the dose-response curves of isoprenaline displacement immediately after addition of the ligands.

*K*_*i*_^*app*^ were calculated using the Cheng-Prusoff equation (2):

2 $${K}_{i}^{app}=\,\frac{IC50}{\frac{[A]}{EC50}+1}$$

where [A] – concentration of the isoprenaline; IC_50_ – fitted half maximal inhibitory concentration of labeled antagonist; EC_50_ – fitted half maximal response concentration of isoprenaline.

### Receptor-ligand saturation binding assay (*K*_*d*_ determination)

*K*_*d*_ determination was performed on Tag-lite β_1_AR (Cisbio, Cat. No. C1TT1BETA1) or β_2_AR (Cisbio, Cat. No. C1TT1BETA2) Tb^3+^ cryptate labeled transfected cells. The β_1_AR or β_2_AR cells labeled with SNAP-Terbium interact with the specific ligand labeled with the corresponding fluorophore. The fraction of bound ligand is proportional to the FRET signal. Donor Tb^3+^ cryptate: exc. 340 nm, em. 490 nm, 546 nm, 583 nm, 620 nm; acceptor KK114: exc. 630 nm, em. 655 nm. The cells were thawed and suspended in 4.5 ml Tag-lite buffer (Cisbio, Cat. No. LABMED) according to the product information and transferred to half-area white polystyrene 96 well plates (Sigma, Cat. No. CLS3693) by 20 µl per well. 20 µl of a fluorescent probe or control compound in Tag-lite buffer was added to each well and incubated for 2 h at room temperature (20 °C) before readout. FRET readout was performed on a multiwell plate reader Spark 20 M (Tecan) at room temperature. Nonspecific binding was determined by adding 50 µM carazolol or BI-167107 to the cell suspensions. All probe and control compound dilutions were prepared in DMSO, so that the final DMSO concentration was kept constant throughout the titration series. The ratio of the acceptor and donor emission signals was calculated for each individual well using equation (3):

3 $$Ratio=\frac{{\rm{Signal}}\,655\,{\rm{nm}}-{\rm{Background}}\,{\rm{Signal}}}{{\rm{Signal}}\,620\,{\rm{nm}}-{\rm{Background}}\,{\rm{Signal}}}$$

Luminescence background signal was measured in wells containing 40 µl of Tag-lite buffer only. Specific binding was calculated by subtracting nonspecific binding ratio from total binding ratio at each fluorescent ligand concentration. *K*_*d*_^*app*^ is determined by fitting data using GraphPad Prism 6 to one site specific binding with Hill slope equation (4):

4 $$Specific\,singal\,ratio=\frac{A\ast {[probe]}^{h}}{{({K}_{d}^{app})}^{h}\,+\,{[probe]}^{h}}$$

A is amplitude of specific signal ratio, [probe] - probe concentration, *K*_*d*_^*app*^ - the ligand concentration needed to achieve a half-maximum binding at equilibrium, h - the Hill slope.

### Cells preparation for imaging experiments

We performed imaging 48–72 h after transfection. Staining of the transfected cell was performed in Hanks’ balanced salt solution (HBSS, Lonza) without Phenol Red for 30–40 min at room temperature (to minimize internalization). Samples were washed two times with HBSS, and cells were imaged in DMEM (Thermo Fisher Scientific) supplemented with GlutaMAX-1 (Thermo Fisher Scientific) and 10% fetal bovine serum (FBS). For all staining experiments with cells, stock solutions of dyes in DMSO were used, and final DMSO content was < 0.5%. Imaging was performed on Leica SP8 (Leica Microsystems) or Abberior STED (Abberior Instruments GmbH) microscopes.

In no-wash experiments, the U2OS cells expressing β_2_AR-YFP fusion protein were incubated in DMEM growth media with 5 nM ab118171 or 100 nM carazolol-KK114 (**24**) for 90 min before imaging on Leica SP8 (Leica Microsystems, Mannheim, Germany) without washing off the excess of probe.

Competitive displacement experiment was performed in living U2OS cells expressing β_2_AR-YFP fusion protein. The cells were incubated with a mixture of fluorescent ligand (5 nM solution of ab118171 or 100 nM solution of other ligands) and 10 mM BI-167107 or carazolol in presence of 0.1 µg/ml Hoechst 33342 for 30 min and imaged on a Leica SP8 (Leica Microsystems, Mannheim, Germany).

For staining of CAPAN-1 cells, fluorescent probes or mixtures of a probe and a competitor were incubated in McCoy’s 5a Medium Modified supplemented with 10% fetal bovine serum (FBS) for 30–40 min at room temperature. Afterwards, the samples were washed two times with HBSS, and cells were imaged in DMEM (Thermo Fisher Scientific) supplemented with GlutaMAX-1 (Thermo Fisher Scientific) and 10% fetal bovine serum (FBS). Imaging performed on Leica SP8 (Leica Microsystems) or Abberior STED (Abberior Instruments GmbH) microscopes.

### Microscopy setups

Confocal and STED images were acquired with Abberior STED 775 QUAD scanning microscope (Abberior Instruments GmbH) equipped with 488 nm, 561 nm and 640 nm 40 MHz pulsed excitation lasers, a pulsed 775 nm 40 MHz STED laser, and UPlanSApo 100x/1.40 Oil objective. Pixel size was 30–40 nm for all STED images acquired on this setup. Laser powers were optimized for each sample.

Confocal imaging was performed on a Leica SP8 (Leica Microsystems, Mannheim, Germany) inverted confocal microscope equipped with an HC PL APO CS2 63x/1.40 Oil objective. Images were acquired using a 700 Hz bidirectional scanner, pixel size of 70 nm × 70 nm, pinhole of 95.6 µm diameter(1 AU) and frame averaging of 2. Hoechst 33342 was excited with 405 nm laser and detected with a regular PMT in the 425–470 nm range. β_2_AR-YFP was excited with a 488 nm laser and detected with a regular PMT in the 525–565 nm range. β_2_AR probes were excited with 633 nm laser and detected with the Leica HyD detector set within the spectral range of 650–750 nm.

All acquired or reconstructed images were processed and visualized using Fiji software (see Supplementary Results)^50^.

### Statistical analysis

Results are expressed as the means ± standard deviation (SD).The statistical data analysis was done using GraphPad Prism 6 software (GraphPad Software, Inc.). Technical replicates from each experiment were averaged and the obtained value was considered obtained from a single independent experiment. All independent experiments were performed in triplicate unless stated differently. Each independent experiment involving cells was performed on a different day using a different passage of the cell lines. For imaging experiments, at least three fields of view were randomly acquired and only one representative field of view from each experiment is shown.

### Data availability

All data generated or analyzed during this study are included in this published article (and its Supplementary Information files).

## Electronic supplementary material

SUPPLEMENTARY INFO
