# Supplementary material for: High-Affinity Functional Fluorescent Ligands for Human β-Adrenoceptors
Source: Sci Rep. 2017 Sep 26;7:12319. doi: 10.1038/s41598-017-12468-3 (PMC5614969; doi:10.1038/s41598-017-12468-3)
Supplement: Supplementary file 1 — SUPPLEMENTARY INFO [file 41598_2017_12468_MOESM1_ESM.pdf]

Supporting information

High-Affinity Functional Fluorescent Ligands for Human β-Adrenoceptors

Gyuzel Y. Mitronova[1*#], Gražvydas Lukinavičius[1*#], Alexey N. Butkevich[1], Tobias Kohl[2], Vladimir N. Belov[1*], Stephan E. Lehnart[2,3] and Stefan W. Hell[1,3]

[1] Department of NanoBiophotonics, Max Planck Institute for Biophysical Chemistry, Am Fassberg 11, 37077 Göttingen, Germany

[2] Heart Research Center Göttingen, Department of Cardiology & Pulmonology, University Medical Center Göttingen, Robert-Koch-Str 40, 37075 Göttingen, Germany

[3] German Center for Cardiovascular Research (DZHK), partner site Göttingen, Germany

[#] these authors contributed equally.

[*] Corresponding authors: E-mail: gmitron@gwdg.de, glukina@gwdg.de, vbelov@gwdg.de.

Contents

Supplementary Notes 4
Chemical synthesis 4
Compound 3 4
Compound 4 5
Compound 5 5
Compound 7 6
Compound 8 6
Compound 9 7
Compound 12 7
Compound 13 8
Compound 14 8
Compound 15 9
Compound 16 9
Compound 17 10
Compound 18 10
Compound 19 11
Benzyl protected BI-167107 11
BI-167107 11
Compound 20 12
Compound 21 12
Compound 22 12
Compound 23 13
Compound 24 13
Supplementary Results 15
Supplementary Tables 15
Supplementary Table 1. Spectral properties of the dyes of the present study. 15
Supplementary Table 2. Properties of fluorescent β_2AR ligands 15
Supplementary Table 3. Quantum yields in PBS pH 7.4 buffer 15
Supplementary Table 4. Fitted full width at half maximum (FWHM) value of profiles shown in figure 7 15
Supplementary Figures 16
Supplementary Figure 1. Spectra of fluorophores used in this study. 16
Supplementary Figure 2. Intracellular cAMP sensor response to β-AR agonists 17

Supplementary Figure 3. Dose response curves of βAR agonist based probes in HEK 293 cells expressing 3',5'-cAMP FRET sensor....18
Supplementary Figure 4. Intracellular cAMP sensor response to competitive displacement of isoprenaline with βAR antagonists in HEK293 cells expressing biosensor $^{T}Epac^{VV}$....19
Supplementary Figure 5. Saturation binding assay on cells expressing β_1ARs or β_2ARs labelled with Lumi4-Tb cryptate....20
Supplementary Figure 6. Confocal images of live U2OS cells expressing β_2AR-YFP fusion protein and stained with 610CP ligands....21
Supplementary Figure 7. Competitive binding experiment: BI-PEG-BDY630 (17)....22
Supplementary Figure 8. Competitive displacement of ab118171 and carazolol KK114 (24) with parent ligand - carazolol....23
Supplementary Figure 9. Excitation and emission spectra of fluorescent dyes under different buffer conditions....24
Supplementary Figure 10. No-wash images of living U2OS cells expressing β_2AR-YFP fusion protein....25
Supplementary Figure 11. Fluorescence emission spectra of the probes in phosphate buffer at different pH values....26
Supplementary Figure 12. Evaluation of confocal and STED images of living U2OS cells expressing β_2AR-YFP stained with fluorescent ligands....27
Supplementary Figure 13 Summary of fluorescent ligands properties used for staining of living cells....28
Supplementary Figure 14. STED images of living CAPAN-1 cells stained with fluorescent ligands....29
Fiji image processing script....30
Supplementary abbreviations....31
Supplementary references....32

Supplementary Notes

Chemical synthesis

BDB synthesis was performed as described by Bonnert et al.[1] with the last step following the procedure by Floyd et al.[2]

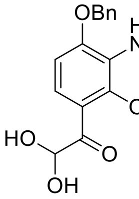

BDB

The synthesis of methyl 2-(3-bromo-2-methylphenyl)acetate was performed according to the procedure described by Winum et al.[3] starting from 3-bromo-2-methylbenzoic acid (TCI). The synthesis of 1-(3-bromo-2-methylphenyl)-2-methylpropan-2-amine (**1**) was performed following the procedure of Glossop et al.[4]

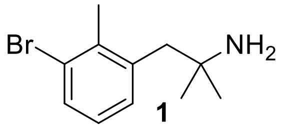

1

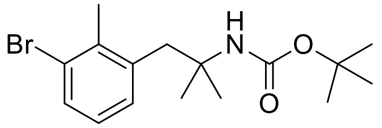

Compound 2. 118 mg (0.49 mmol) of **1-(3-bromo-2-methylphenyl)-2-methylpropan-2-amine** (**1**) and 120 mg (0.55 mmol) of Boc_2O were dissolved under 0 ˚C in dry THF (2.5 ml). The obtained solution was added gradually into another flask (at –20 ˚C or lower temperature) containing NaHMDS (0.46 ml of 1.9 M THF solution, ~0.87 mmol,). The reaction mixture was stirred at below –20 ˚C for 45 min. Then cold (0 ˚C) sodium citrate buffer (pH 4, 20 ml) was added gradually to the reaction mixture. The reaction mixture was stirred for another 15 min at 0 ˚C. After warming up to the room temperature Et_2O (20 ml) was added. The organic layer was separated, and the aqueous layer was extracted with ether (2×7 ml). The combined organic solutions were washed with brine (10 ml), dried, and the solvent was evaporated in vacuo. TLC, hexane/EtOAc 2:1, R_f = 0.7; or in pure hexane R_f = 0.2. The product was isolated by flash column chromatography (Biotage SNAP Ultra 10 g; gradient 0% to 30% of ethyl acetate in hexane). Yield – 125 mg (75%) of yellowish solid.

^{1}H NMR (400 MHz, $CDCl_3$): δ = 7.44 (dd, $^3J_{H,H}$ = 7.7, $^4J_{H,H}$ = 1.4, 1H, H-6 or H-4), 7.06 (dd, $^3J_{H,H}$ = 7.7, $^4J_{H,H}$ = 1.4, 1H, H-4 or H-6), 6.95 (t, $^3J_{H,H}$ = 7.8, 1H, H-5), 4.35 (s, 1H, NH), 3.12 (s, 2H, CH_2), 2.42 (s, 3H, CH_3), 1.47 (s, 9H, CH_3), 1.25 (s, 6H, CH_3).

^{13}C NMR (101 MHz, $CDCl_3$): δ = 154.6 (C=O), 138.9 (C), 137.1 (C), 131.1 (CH), 131.0 (CH), 126.5 (CH), 126.5 (C), 54.1 (C_q-N), 42.2 (CH_2), 28.7 (CH_3-Boc), 27.8 (CH_3), 20.8 (CH_3); C_q-Boc signal overlaps with chloroform.

ESI-MS, positive mode: *m/z* (rel. int., %) = 705/707/709 $[2M+Na]^+$, 364/366 $[M+Na]^+$.

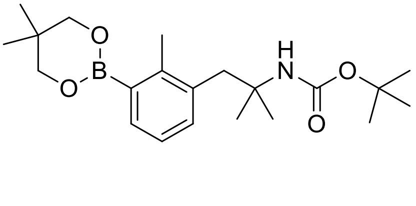

Compound 3. A mixture of bromide **2** (200 mg, 0.58 mmol), $Pd(dppf)Cl_2 \cdot CH_2Cl_2$ (29 mg, 0.04 mmol, 7 mol %), KOAc (298 mg, 3.04 mmol), bis(neopentyl glycolato)diboron (190 mg, 0.84 mmol) in 1,4-dioxane (2 ml) was degassed with a stream of Ar for 10 min and then stirred at 110 ˚C for 3 h in a screw-cap tube. The reaction mixture

was filtered through Celite, and the filter cake was washed with Et_2O (2×50 ml). The organic solution was washed with sodium citrate buffer and brine, dried over anhydrous Na_2SO_4, filtered and concentrated to dryness. The residue was purified by column chromatography on SiO_2 (n-pentane/EtOAc 20:1). Yield – 159 mg (73%) of white solid.

^{1}H NMR (500 MHz, DMSO-d_6): δ = 7.39 (dd, J = 7.3, 1.6, 1H, H-4 or H-6), 7.08 (dd, J = 7.3, 1.6, 1H, H-6 or H-4), 7.02 (t, J = 7.3, 1H, H-5), 6.35 (br.s. 1H, NH), 3.74 (s, 4H, CH_2O), 2.97 (s, 2H, CH_2), 2.40 (s, 3H, CH_3), 1.41 (s, 9H, CH_3), 1.13 (s, 6H, CH_3), 0.97 (s, 6H, CH_3).

^{13}C NMR (126 MHz, DMSO-d_6): δ = 154.3 (C=O), 141.4 (C), 136.5 (C), 133.1 (CH), 132.2 (CH), 124.0 (CH), 77.1(C_q-Boc), 71.5 (C_2O), 54.2 (C_q-N), 40.1 (CH_2, overlaps with DMSO-d_6 signal), 31.2 (C), 28.4 (CH_3-Boc), 27.2 (CH_3), 21.3 (CH_3), 19.0 (CH_3); C–B signal is not observed due to quadrupolar relaxation.

ESI-MS, positive mode: m/z (rel. int., %) = 398 $[M+Na]^+$.

Compound 4. Compound **3** (355 mg, 0.95 mmol) was dissolved in *n*-butanol (15 ml) and ***trans*-ethyl-3-bromoacrylate**[5] (423 mg, 2.36 mmol) was added. The mixture was degassed with a stream of Ar. Then $Pd(dba)_2$ (44 mg, 0.08 mmol), SPhos (31 mg, 0.08 mmol), K_3PO_4 (604 mg, 2.85 mmol) and 4 ml of degassed water were added sequentially. The mixture was stirred at 100 ˚C for 20 h, cooled to room temperature, and then water (15 ml) and Et_2O (15 mL) were added. The organic layer was separated. The aqueous layer was extracted with Et_2O (2×10 mL); combined organic layers were washed with water and brine (10 ml), dried over Na_2SO_4 and then the solvents were evaporated in vacuo. The residue was purified by column chromatography on SiO_2 (n-pentane/EtOAc 20:1). Yield – 243 mg (71%) of white solid.

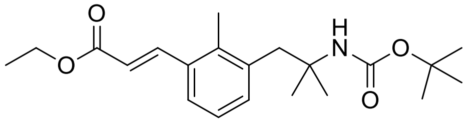

^{1}H NMR (400 MHz, $CDCl_3$): δ = 8.05 (d, J = 15.8, 1H, CH), 7.41 (dd, J = 7.3, 1.9 Hz, 1H, H-arom), 7.18 – 7.09 (m, 2H, H-arom), 6.30 (d, J = 15.8, 1H, CH), 4.34 (s, 1H, NH), 4.27 (q, J = 7.1 Hz, 2H, $\mathbf{CH_2}CH_3$), 3.10 (s, 2H, CH_2), 2.40 (s, 3H, CH_3), 1.47 (s, 9H, Boc), 1.34 (t, J = 7.1, 3H, $CH_2\mathbf{CH_3}$), 1.26 (2×s, 6H, CH_3).

^{13}C NMR (101 MHz, $CDCl_3$): δ = 171.3 (C=O), 167.0 (C=O), 143.8 (CH), 137.6 (C), 136.8 (C), 134.6 (C), 133.8 (CH), 125.5 (CH), 125.2 (CH), 119.9 (CH), 77.1 (C_q-Boc, overlaps with $CDCl_3$ signal), 60.6 (CH_2O), 54.1 (C_q-N), 41.4 (CH_2), 28.7 (CH_3), 27.8 (CH_3), 16.6 (CH_3), 14.5 (CH_3).

ESI-MS, positive mode: m/z (rel. int., %) = 384/386 (100) $[M+Na]^+$.

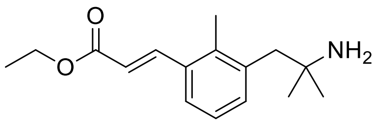

Compound 5. 240 mg (0.66 mmol) of **4** was dissolved in 3 ml DCM, and the solution was cooled to 0 °C. Then 1:1 mixture of TFA and DCM (0.6 ml) was added, and the reaction mixture was stirred overnight at rt. After diluting with DCM, washing with sat. aq. $NaHCO_3$ and drying over Na_2SO_4, the solvent was evaporated in vacuo. Yield – 166 mg (97%) of clear viscous oil.

^{1}H NMR (400 MHz, $CDCl_3$): δ = 8.04 – 7.94 (m, 1H, CH), 7.45 (dd, J = 7.3, 1.8, 1H), 7.21 – 7.10 (m, 2H), 6.30 (d, J = 15.8, 1H, CH), 4.26 (q, J = 7.1, 2H, CH_2O), 3.10 (s, 2H, CH_2), 2.39 (s, 3H, CH_3), 2.04 (s, 2H, NH_2), 1.39 – 1.29 (m, 9H, CH_3).

^{13}C NMR (101 MHz, $CDCl_3$): δ = 167.0 (C=O), 143.1, 136.7, 135.4, 134.1, 133.3, 126.4, 126.1, 120.7, 60.7, 56.5, 42.1, 25.0, 16.5, 14.3.

ESI-MS, positive mode: *m/z* (rel. int., %) = 262 (100) $[M+H]^+$.

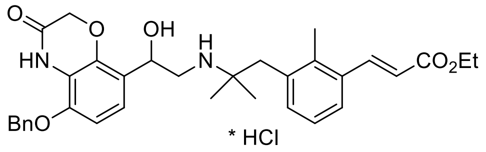

Compound 7. Amine **5** (140 mg, 0.54 mmol) was dissolved in ethanol (1.4 ml), then **BDB** (160 mg, 0.49 mmol) was added at 70˚C. The mixture was stirred for 15 minutes and cooled to room temperature. The precipitate was dissolved by adding 3.4 ml THF to the reaction mixture. After the reaction mixture was cooled to 0˚C, 30 mg (0.79 mmol) of $NaBH_4$ was added. The reaction mixture was then warmed up to room temperature and stirred for one hour; acetone (6 ml) was added, and stirring was continued for further 30 min. The reaction mixture was diluted with ethyl acetate (12 ml) and washed with water (6 ml). The organic layer was dried over sodium sulfate, filtered and evaporated in vacuo. The residue was dissolved in 4 ml of MeOH/EtOAc mixture (3:1), and 4 mL of 1 M aq. HCl was added. The product precipitated upon addition of Et_2O (8 ml). The precipitate was filtered off and washed several times with water. Yield – 166 mg (61%) of light-brown solid. HPLC: t_R = 15.6 min (A/B: 10/90 – 100/0 in 25 min, 1.2 ml/min, 254 nm).

^1H NMR (400 MHz, DMSO-d_6): δ = 10.13 (s, 1H, NH), 7.96 (d, *J* = 15.8, 1H, CH), 7.57 – 7.49 (m, 3H), 7.41 – 7.34 (m, 2H), 7.34 –7.28 (m, 1H), 7.22 – 7.17 (m, 1H), 7.11 (t, *J* = 7.7, 1H), 6.97 (d, *J* = 8.8, 1H), 6.75 (d, *J* = 8.8, 1H), 6.42 (d, *J* = 15.8, 1H, CH), 5.17 (s, 2H, CH_2O), 4.78 (d, *J* = 8.8, 1H, CH), 4.53 (d, AB system, *J* = 14.9, 1H, CH_2O), 4.47 (d, AB system, *J* = 14.9, 1H, CH_2O), 4.19 (q, *J* = 7.1, 2H, $\mathbf{CH_2}CH_3$), 3.17 (d, *J* = 5.1, 2H, NCH_2), 2.76 – 2.63 (m, 2H, CH_2), 2.34 (s, 3H, CH_3), 1.26 (t, *J* = 7.1, 3H, $CH_2\mathbf{CH_3}$), 0.95 (s, 6H, CH_3).

^{13}C NMR (101 MHz, DMSO-d_6): δ = 166.1 (C=O), 164.4 (C=O), 145.4, 142.7, 141.1, 136.9, 136.8, 134.8, 134.1, 133.8, 128.3, 127.7, 127.6, 125.8, 125.6, 122.0, 120.1, 119.8, 116.7, 106.8, 69.7, 67.0, 63.4, 60.6, 60.1, 46.8, 22.1, 22.0, 16.3, 14.2. Two C signals are not resolved.

ESI-MS, positive mode: m/z (rel. int., %) = 559 (100) $[M+H]^+$.

HRMS (*m/z*): $[M+H]^+$ calcd. for $C_{33}H_{38}N_2O_6$, 559.2803; found, 559.2806.

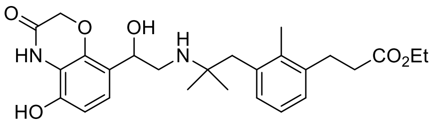

Compound 8. A 50 ml Schlenk flask was evacuated and flushed with argon two times. 10% Pd/C (60 mg; Merck, oxidized form) and THF (4 ml) were added, and the mixture was stirred vigorously under hydrogen to activate the catalyst. A solution of **7** (166 mg, 0.30 mmol) in 6 ml of EtOH/MeOH mixture (4:1) was then added. The solution was stirred overnight at 20 °C under H_2. Hydrogen was replaced with argon, and the mixture was filtered through Celite. The filter cake was washed with EtOAc and MeOH. The solvents were evaporated in vacuo. The title compound was purified by chromatography on SiO_2 (40 g) with CH_2Cl_2/MeOH mixture (5:1, 0.1% NH_4OH). Yield – 133 mg (95%) of light-brown solid. HPLC: t_R = 11.9 min (A/B: 10/90 – 100/0 in 25 min, 1.2 ml/min, 254 nm).

^1H NMR (400 MHz, CD_3OD): δ = 7.16 – 7.02 (m, 4H), 6.59 (d, *J* = 8.5, 1H), 5.20 (dd, *J* = 9.9, 2.7, 1H, CH), 4.65 (d, AB system, *J* = 14.9, 1H, CH_2O), 4.58 (d, AB system, *J* = 14.9, 1H, CH_2O), 4.10 (q, *J* = 7.1, 2H, $\mathbf{CH_2}CH_3$), 3.35 – 3.32 (m, 1H, H^D part of the CH^CH^DN), 3.20 – 3.06 (m, 3H, H^C part of the CH^CH^DN,

CH_2), 2.97 (dd, *J* = 8.3, 7.1, 2H, CH_2), 2.59 (dd, *J* = 8.3, 7.1, 2H, CH_2), 2.35 (s, 3H, CH_3), 1.29 (2×s, 6H, CH_3), 1.21 (t, *J* = 7.1, 3H, CH_2**CH_3**).

^{13}C NMR (101 MHz, DMSO-d_6): δ = 172.3, 164.4, 144.0, 141.2, 138.8, 137.0, 135.0, 129.7, 126.5, 124.6, 122.9, 120.0, 115.1, 108.9, 66.9, 66.3, 59.8, 55.3, 48.9, 43.0, 34.2, 28.7, 26.5, 15.6, 14.1.

ESI-MS, positive mode: m/z (rel. int., %) = 471 (100) $[M+H]^+$.

HRMS (*m/z*): $[M-H]^-$calcd. for $C_{26}H_{34}N_2O_6$, 469.2344; found, 469.2335.

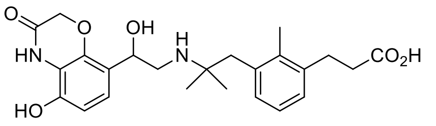

Compound 9. Ethyl ester **8** (106 mg, 0.33 mmol) was dissolved in 3 ml EtOH, the solution was cooled down to 0˚C and 1.2 ml of 1 M NaOH was added. After stirring at room temperature for 3 h, the reaction mixture was neutralized with 1 M aq. HCl, and the solvents were evaporated *in vacuo*. The title compound was isolated by reversed phase chromatography on RP-C_{18} with MeCN/H_2O + 0.1% HCOOH mixture (1:1). Yield − 142 mg (98 %) of brownish solid, obtained upon freeze-drying. HPLC: t_R = 8.6 min (A/B: 10/90 – 100/0 in 25 min, 1.2 ml/min, 254 nm).

^{1}H NMR (400 MHz, DMSO-d_6): δ = 8.38 (s, 1H), 7.07 – 6.89 (m, 4H), 6.53 (d, *J* = 8.5, 1H), 4.92 (dd, *J* = 9.9, 2.7, 1H, CH), 4.53 (d, AB system, *J* = 14.9, 1H, CH_2O), 4.47 (d, AB system, *J* = 14.9, 1H, CH_2O), 2.86 – 2.93 (m, 1H, N-CH_2), 2.76 – 2.86 (m, 4H, CH_2, N-CH_2), 2.68 (t, *J* = 7.8, 1H, N-CH_2), 2.32 (t, *J* = 7.8, 2H, CH_2), 2.24 (s, 3H, CH_3), 1.03 (s, 6H, CH_3).

^{13}C NMR (101 MHz, DMSO-d_6): δ = 174.6, 164.4, 144.4, 141.2, 139.8, 135.7, 135.1, 129.6, 126.8, 124.7, 121.8, 120.0, 115.2, 109.1, 66.9, 65.1, 56.5, 48.1, 41.3, 35.0, 29.1, 24.4, 15.6.

ESI-MS, positive mode: m/z (rel. int., %) = 443 (100) $[M+H]^+$.

HRMS (*m/z*): $[M+H]^+$ calcd. for $C_{24}H_{30}N_2O_6$, 443.2177; found, 443.2178.

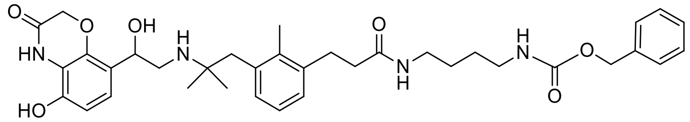

Compound 12. Carboxylic acid **9** (50 mg, 0.11 mmol) was dissolved in DMF (1 ml). DIEA (30 µl, 0.17 mmol) and PyBOP (125 mg, 0.24 mmol) were successively added. After 5 min, *N*-carbobenzoxy-1,3-diaminobutane hydrochloride (TCI, 97%, 39 mg, 0.15 mmol) was added, and the reaction mixture was stirred for 1 h at room temperature. The title compound was isolated by chromatography on SiO_2 (25 g) with CH_2Cl_2/MeOH mixture (5:1, 0.1% NH_4OH) as an eluent. Yield – 66 mg (93%) of light-brown solid.

^{1}H NMR (400 MHz, DMSO-d_6): δ = 10.01 (s, 1H, NHCO), 7.85 (t, *J* = 5.6, 1H, $NHCOCH_2$), 7.43 – 7.15 (m, 5H, C_6H_5), 7.07 – 6.99 (m, 3H, H_{ar}), 6.97 (d, *J* = 8.5, 1H), 6.60 (d, *J* = 8.5, 1H), 6.00 (br. s, 1H, OH), 5.15 (d, *J* = 9.9, 1H, CH), 4.99 (s, 2H, CH_2O), 4.61 (d, AB system, *J* = 14.9, 1H, CH_2O), 4.53 (d, AB system, *J* = 14.9, 1H, CH_2O), 4.12 (q, *J* = 5.3, 1H), 3.18 – 3.06 (m, 5H), 3.01 – 2.92 (m, 4H, CH_2), 2.79 (t, *J* = 8.0, 2H, CH_2), 2.30 – 2.22 (m + s, 2+3H, CH_2, CH_3), 1.43 – 1.28 (m, 4H, CH_2), 1.18 (2×s, 6H, CH_3).

^{13}C NMR (101 MHz, DMSO-d_6): δ = 171.2, 164.3, 156.1, 144.9, 141.3, 140.4, 137.3, 135.5, 134.1, 130.0, 129.8, 127.7, 127.4, 125.0, 120.1, 120.0, 115.3, 114.7, 109.2, 67.0, 65.1, 63.5, 60.7, 48.6, 47.0, 41.5, 38.1, 35.9, 29.5, 26.9, 26.5, 22.2, 22.1, 15.8.

ESI-MS, positive mode: m/z (rel. int., %) = 647 (100) $[M+H]^+$; 669 (10) $[M+Na]^+$.

HRMS (*m/z*): $[M+H]^+$ calcd. for $C_{36}H_{46}N_4O_7$, 647.3439; found, 647.3439.

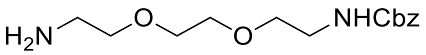

Linker **11** was prepared from 10 g (67 mmol) of 1,2-bis(2-aminoethoxy)ethane (Alfa Aesar, 98%) and 3.0 g (17.6 mmol) benzyl chloroformate (Alfa Aesar, 95%, stab. with ca 0.1% sodium carbonate) according to the literature procedure.[6] Yield – 2.5 g, 50%.

Compound 13 was prepared from 34 mg (0.12 mmol) of **11**, 30 mg (0.07 mmol) **9** and 62 mg (0.12 mmol) PyBOP in 1 ml DMF accoding to the method described above for the compound **12**. After the solvent was evaporated *in vacuo*, the product was isolated by flash column chromatography (Biotage SNAP Ultra 10 g; gradient 2% to 80% MeOH in DCM + 0.1% NH_4OH). Yield – 32 mg (64%). HPLC: t_R = 8.6 min (A/B: 30/70 – 100/0 in 25 min, 1.2 ml/min, 254 nm).

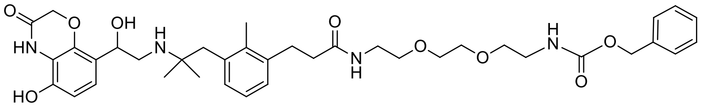

^{1}H NMR (400 MHz, CD_3OD): δ = 7.36 – 7.21 (m, 5H, Ph), 7.13 – 6.98 (m, 4H), 6.57 (d, J = 8.5, 1H), 5.14 (dd, J = 9.5, 3.2, 1H, CHO), 5.03 (s, 2H, CH_2O), 4.58 (d, AB system, J = 14.9, 1H, CH_2), 4.52 (d, AB system, J = 14.9, 1H, CH_2), 3.60 – 3.44 (m, 8H, CH_2), 3.37 – 3.25 (m, 4H, CH_2, overlaps with CD_3OD signal), 3.17 (dd, J = 12.1, 3.3, 1H, H^D part of the CH^CH^DN), 3.08 – 2.97 (m, 3H, H^C part of the CH^CH^DN, CH_2), 2.96 – 2.87 (m, 2H, CH_2), 2.49 – 2.39 (m, 2H, CH_2), 2.31 (s, 3H, CH_3), 1.21 (s, 3H, CH_3), 1.20 (s, 3H, CH_3).

^{13}C NMR (101 MHz, CD_3OD): δ = 175.3, 166.7, 158.8, 146.7, 142.7, 141.5, 138.3, 136.6, 135.7, 131.2, 129.4, 129.1, 129.0, 128.8, 127.4, 127.3, 126.5, 121.9, 121.5, 116.3, 110.2, 71.3, 71.3, 70.9, 70.6, 68.1, 67.4, 66.3, 60.6, 42.2, 41.7, 40.8, 40.3, 37.6, 31.1, 24.3, 24.1, 16.4.

ESI-MS, positive mode: *m/z* (rel. int., %) = 707 (100) $[M+H]^+$, 729 (40) $[M+Na]^+$.

HRMS (*m/z*): $[M+H]^+$ calcd. for $C_{38}H_{50}N_4O_9$, 707.3651; found, 707.3657.

Compound 14. A 50 ml Schlenk flask was evacuated and flushed with argon. 10% Pd/C (75 mg; Merck, oxidized form) and THF (4 ml) were added, and the mixture was stirred vigorously under hydrogen to activate the catalyst. A solution of **12** (75 mg, 0.12 mmol) in 5 mL THF/MeOH mixture (10:1) was injected into the flask through a rubber septum. The solution was stirred overnight at 20 °C under H_2. Then hydrogen was replaced by argon, and the mixture was filtered through Rotilabo PTFE syringe filter (0.2 µm). The solvents were evaporated in vacuo. The title compound was isolated by chromatography on RP-C_{18} (20 g) with MeCN/H_2O mixture (1:2, 0.1% HCOOH) as an eluent. Yield – 58 mg (94%) of light-brown solid. HPLC: t_R = 10.1 min (MeCN/H_2O + 0.05M TEAB: 10/90 – 70/30 in 20 min, 1.0 ml/min, 254 nm).

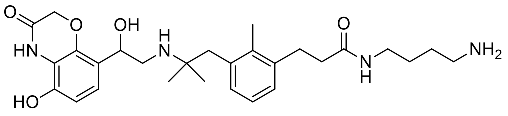

^{1}H NMR (600 MHz, DMSO-d_6): δ = 8.32 (s, 1H, NHCO), 7.84 (s, 1H, NHCO), 7.05 – 6.96 (m, 3H), 6.93 (d, J = 8.4, 1H), 6.54 (d, J = 8.4, 1H), 4.93 (d, J = 7.9, 1H, CHO), 4.53 (d, AB system, J = 14.9, 1H, CH_2), 4.49 (d, AB system, J = 14.9, 1H, CH_2), 3.05 (d, J = 6.2, 2H, CH_2), 2.94 – 2.70 (m, 8H, CH_2), 2.30 (t, J = 8.0, 2H, CH_2), 2.25 (s, 3H, CH_3), 1.53–1.47 (m, 2H, CH_2), 1.46–1.40 (m, 2H, CH_2), 1.05 (s, 6H, CH_3).

^{13}C NMR (126 MHz, DMSO-d_6): δ = 171.4 (CO), 164.4 (CO), 144.6 (C), 141.2 (C), 140.1 (C), 135.4 (C), 135.2 (C), 129.7 (CH), 127.0 (CH), 124.9 (CH), 121.4 (C), 120.0 (CH), 115.2 (C), 109.0 (CH), 67.0 (C), 66.4 (C/CH_2), 64.7 (CH), 47.8 (C/CH_2), 38.6 (CH_2), 37.8 (CH_2), 36.0 (CH_2), 29.6 (CH_2), 26.2 (CH_2), 24.7 (CH_2), 24.2 (CH_3), 23.9 (CH_3), 15.7 (CH_3).

ESI-MS, positive mode: *m/z* (rel. int., %) = 513 (100) $[M+H]^+$; 535 (40) $[M+Na]^+$.

HRMS (*m/z*): $[M+H]^+$ calcd. for $C_{28}H_{40}N_4O_5$, 513.3071; found, 513.3067.

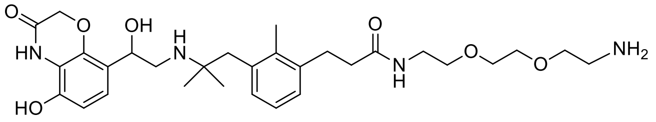

Compound 15 was prepared as described for compound **14** from 27 mg (0.04 mmol) **13** and 10 % Pd/C (30 mg; Merck, oxidized form). The product was isolated on RP-C_{18} (10 g) with MeCN/H_2O mixture (1:2, 0.1% HCOOH) as an eluent. Yield – 21 mg (92%) of white powder. HPLC: t_R = 9.0 min, (A/B: 10/90 – 100/0 in 25 min, 1.2 ml/min, 254 nm).

^1H NMR (400 MHz, CD_3OD): δ = 7.08 – 6.90 (m, 4H), 6.52 (dd, J = 8.5, 1.8, 1H), 5.01 (dd, J = 8.2, 2.7, 1H, CHO), 4.55 – 4.42 (m, 2H, CH_2), 3.67 – 3.53 (m, 6H, CH_2), 3.50 (t, J = 5.6, 2H, CH_2), 3.34 (t, J = 5.6, 2H, CH_2), 2.96 – 2.81 (m, 8H, CH_2), 2.46 – 2.38 (m, 2H, CH_2), 2.30 (s, 3H, CH_3), 1.09 (s, 6H, CH_3).

^{13}C NMR (101 MHz, CD_3OD): δ = 175.5, 166.9, 147.1, 142.9, 141.1, 137.5, 136.4, 131.0, 128.5, 126.2, 122.2, 122.1, 116.3, 110.5, 71.5, 71.3, 71.3, 70.6, 68.1, 68.0, 56.8, 44.0, 41.6, 40.3, 37.7, 31.2, 26.2, 25.9, 16.4.

ESI-MS, positive mode: *m/z* (rel. int., %) = 573 (100) $[M+H]^+$, 595 (40) $[M+Na]^+$.

HRMS (*m/z*): $[M+H]^+$ calcd. for $C_{30}H_{44}N_4O_7$, 573.3283; found, 573.3284.

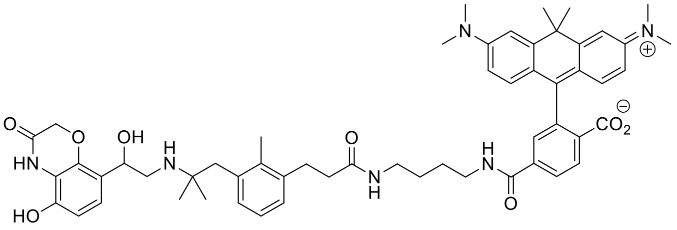

Compound 16. Amine **14** (10 mg, 19.5 µmol) was dissolved in 0.1 ml DMF. Dye 610CP[7] (NHS ester, 2.0 mg, 3.6 µmol) in 50 µl DMF and 5 µl Et_3N were added, and the reaction mixture was stirred at room temperature for 30 min under argon. The solvent was evaporated *in vacuo*, and the product was isolated on SiO_2 (30 g) using MeCN/H_2O + 0.1% HCOOH (3:1) mixture as an eluent. Yield – 0.9 mg (26%) of dark blue solid. HPLC: t_R = 6.6 min (MeCN/H_2O + 0.05% TFA: 30/90 – 100/0 in 20 min, 1.2 ml/min, 636 nm).

ESI-MS, positive mode: *m/z* (rel. int., %) = 951 (100) $[M+H]^+$.

HRMS (*m/z*): $[M+2H]^{2+}$ calcd. for $C_{56}H_{66}N_6O_8$, 476.2544; found, 476.2539.

HRMS (*m/z*): $[M+H]^+$ calcd. for $C_{57}H_{68}N_6O_8$, 965.5171; found, 965.5157.

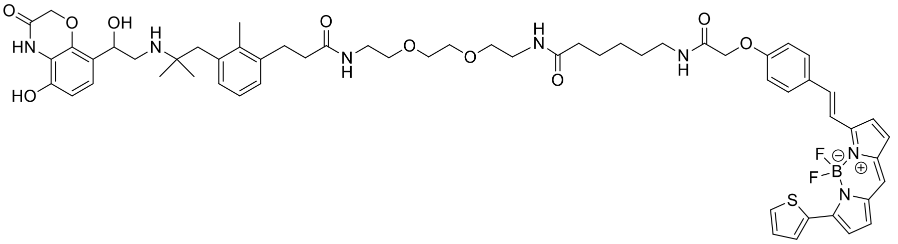

Compound 17. Amine **15** (4 mg, 6.8 µmol) was dissolved in DMF (1 ml). Commercially available amino-reactive fluorophore 6-(((4,4-difluoro-5-(2-thienyl)-4-bora-3a,4a-diaza-s-indacen-3-yl)styryloxy)acetyl)aminohexanoic acid, succinimidyl ester (BDY630-X, SE, Tocris, 2 mg, 3.0 µM) and Et_3N (5 µl, 36.1 µmol) were added. The reaction mixture was stirred for 1 h at room temperature. The product was isolated by flash column chromatography on RP-C_{18} (10 g, gradient 5% to 100% $MeCN/H_2O$ + 0.1% HCOOH). Fractions containing the product were evaporated, the residue was redissolved in MeOH, filtered through a 0.22 µm PTFE membrane filter and evaporated to dryness. Yield – 1.5 mg (19%) of dark blue solid. HPLC: t_R = 5.8 min, (A/B: 20/80 – 100/0 in 25 min, 1.0 ml/min, 600 nm).

^{1}H NMR (400 MHz, DMSO-d_6): δ = 8.15 (t, J = 5.9, 1H, NH), 8.04 (dd, J = 3.8, 1.1, 1H), 7.93 (t, J = 5.5, 1H, NH), 7.83 (dd, J = 5.2, 1.1, 1H), 7.74 (d, J = 16.3, 1H, CH=), 7.62 – 7.59 (m, 3H), 7.45 – 7.35 (m, 2H, H-arom, CH=), 7.32 – 7.23 (m, 3H), 7.07 (d, J = 8.8, 2H), 6.99 – 6.96 (m, 2H), 6.96 – 6.94 (m, 1H), 6.92 – 6.83 (m, 2H), 6.53 (dd, J = 8.4, 1.1, 1H), 5.03 – 4.98 (m, 1H, CH), 4.83 (s, 2H, CH_2), 4.60 – 4.40 (m, 4H, CH_2O), 3.52 – 3.44 (m, 4H, CH_2), 3.40 – 3.34 (m, 4H, CH_2), 3.22 – 3.14 (m, 4H, CH_2), 3.10 (dd, J = 6.7, 6.7, 2H, CH_2), 2.81 – 2.70 (m, 4H, CH_2), 2.34 – 2.26 (m, 2H, CH_2), 2.24 – 2.20 (m, 3H, CH_3), 2.06 (t, J = 7.5, 2H, CH_2), 1.52 – 1.39 (m, 4H, CH_2), 1.27 – 1.16 (m, 2H, CH_2), 0.97 (s, 3H, CH_3), 0.94 (s, 3H, CH_3).

ESI-MS, positive mode: m/z (rel. int., %) = 1119 (100) $[M+H]^+$.

HRMS (m/z): $[M+H]^+$ calcd. for $C_{59}H_{70}BF_2N_7O_{10}S$, 1118.5048; found, 1118.5041.

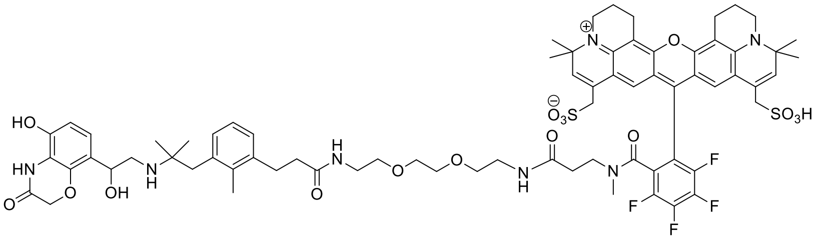

Compound 18 was prepared analogously to compound **17** from **15** (2.8 mg, 4.9 µmol) and KK114[8] (NHS ester, 3.8 mg, 3.9 µmol). After the solvent was evaporated *in vacuo*, the product was isolated by flash column chromatography on amino phase (Biotage SNAP KP-NH 11 g; gradient 7% to 20% MeCN in H_2O + 0.1% HCOOH). Yield – 2.0 mg (35%) of dark blue solid. HPLC: t_R = 7.4 min, (A/B: 20/80 – 100/0 in 20 min, 1.2 ml/min, 630 nm).

^{1}H NMR (500 MHz, CD_3OD): δ = 8.55 (br. s, 1H, NH), 7.45 – 7.27 (m, 2H), 7.15 – 6.99 (m, 3H), 6.56 (dd, J = 8.5, 2.0, 1H), 5.94 – 5.81 (m, 2H), 5.11 (dd, J = 9.9, 2.8, 1H, CH), 4.64 – 4.45 (m, 2H, CH_2), 3.81 – 3.70 (m, 2H, CH_2), 3.69 – 3.52 (m, 8H, CH_2), 3.52 – 3.43 (m, 4H, CH_2), 3.43 – 3.29 (m, 2H, CH_2), 3.06 – 2.82 (m, 9H, CH_2, NCH_3), 2.88 2.79 (m, 2H, CH_2), 2.66 – 2.65 (m, 2H, CH_2), 2.51 – 2.39 (m, 4H, CH_2), 2.35 – 2.22 (m, 3H, CH_3), 2.20 – 2.10 (m, 4H, CH_2), 2.09 – 1.94 (m, 4H, CH_2), 1.62 – 1.48 (m, 12H, CH_3), 1.36 – 1.11 (m, 8H, CH_2, CH_3).

^{13}C NMR (126 MHz, CD_3OD; highly split signals of the fluorinated ^{13}C-atoms were not registered): δ = 175.4, 172.7, 169.3, 166.9, 154.6, 152.24, 152.19, 146.6, 142.7, 142.6, 141.6, 139.0, 138.9, 136.8, 135.5, 131.3, 129.6, 126.6, 124.8, 122.9, 121.8, 121.2, 116.3, 114.3, 110.2, 110.1, 107.8, 107.1, 71.3, 70.6, 68.2, 61.4, 55.1, 54.8, 45.6, 44.6, 41.8, 40.3, 37.6, 31.2, 28.9, 28.4, 23.5, 21.6, 21.4, 16.5.

ESI-MS, negative mode: *m/z* (rel. int., %) = 1441 (100) [*M*-H]$^{-}$.

HRMS (*m/z*): [M+Na]$^{+}$ calcd. for $C_{72}H_{83}F_4N_7O_{16}S_2$, 1464.5166; found, 1464.5149.

Compound 19 was prepared similarly to compound **17**from **15** (4 mg, 7.0 µmol) and 610CP[7] (NHS ester, 2.0 mg, 2.9 µmol) in 0.5 ml DMF. After the solvent was evaporated *in vacuo*, the product was isolated by flash column chromatography on amino phase (Biotage SNAP KP-NH 11 g; gradient 3% to 30% MeCN in H_2O + 0.1% HCOOH). Yield – 1.2 mg (16%) of dark blue solid. HPLC: 100%, t_R = 8.5 min, (A/B: 20/80 – 100/0 in 20 min, 1.2 mL/min, 610 nm).

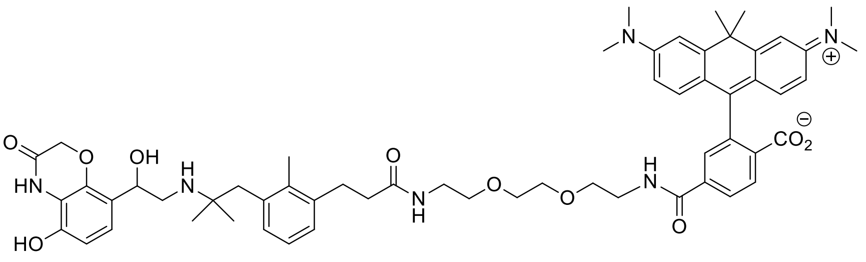

ESI-MS, negative mode: *m/z* (rel. int., %) = 1010 (100) [*M*-H]$^{-}$; ESI-MS, positive mode: *m/z* (rel. int., %) = 1012 (20) [*M*+H]$^{+}$, 506 (100) [*M*+2H]$^{2+}$.

HRMS (*m/z*): [M+H]$^{+}$ calcd. for $C_{58}H_{70}N_6O_{10}$, 1011.5226; found, 1011.5220.

Benzyl protected BI-167107 was prepared similarly to compound **7** from 21 mg (0.13 mmol) of 2-(2-methylbenzyl)propan-2-amine hydrochloride and 38 mg (0.12 mmol) of **BDB**. The product was isolated by flash column chromatography (Biotage SNAP Ultra 10 g; gradient 3% to 20% MeOH in DCM). Yield – 48 mg (87 %) of yellowish solid.

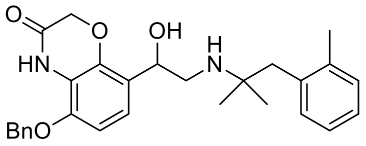

^{1}H NMR (400 MHz, CD_3OD): δ = 7.50 – 7.41 (m, 2H), 7.40 – 7.25 (m, 3H), 7.15 – 7.06 (m, 3H), 7.06 – 6.97 (m, 2H), 6.72 (d, *J* = 8.7, 1H), 5.20 (s, 2H, CH_2O), 5.01 (t, *J* = 8.1, 1H, CHO), 4.54 (d, AB system, *J* = 15.3, 1H, CH_2), 4.49 (d, AB system, *J* = 15.3, 1H, CH_2), 2.86 – 2.78 (m, 2H, CH_2N), 2.76 (s, 2H, CH_2), 2.31 (s, 3H, CH_3), 1.07 (s, 3H, CH_3), 1.06 (s, 3H, CH_3).

ESI-MS, positive mode: *m/z* (rel. int., %) = 461 (100) [*M*+H]$^{+}$.

BI-167107 was prepared by hydrogenation of the benzyl derivative (23 mg, 50 µmol) described above in THF on 10% Pd/C (10 mg; Merck, oxidized form). The product was isolated by flash column chromatography (Biotage SNAP Ultra 10 g; gradient 3% to 80% DCM/MeOH). Yield – 13 mg (70%) of white solid. HPLC: t_R = 3.3 min, (A/B: 10/90 – 100/0 in 20 min, 1.0 ml/min, 600 nm).

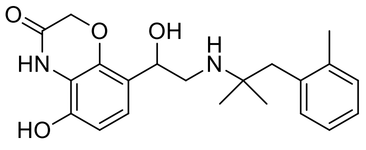

^{1}H NMR (400 MHz, CD_3OD): δ = 7.28 – 7.14 (m, 4H), 7.09 (dd, *J* = 8.5, 0.7, 1H), 6.59 (d, *J* = 8.5, 1H), 5.21 (d, *J* = 7.3, 1H, CH), 4.65 (d, AB system, *J* = 15.1, 1H, CH_2), 4.58 (d, AB system, *J* = 15.1, 1H, CH_2), 3.34-3.36 (m, 1H, H^D part of the CH^CH^DN), 3.16 – 3.02 (m, 3H, H^C part of the CH^CH^DN, CH_2), 2.39 (s, 3H, CH_3), 1.32 (s, 6H, CH_3).

^{13}C NMR (101 MHz, CD_3OD): δ = 166.6 (C=O), 146.7 (C), 142.6 (C), 138.7 (C), 134.4 (C), 132.7 (CH), 132.1 (CH), 128.7 (CH), 126.9 (CH), 121.7 (CH), 120.9 (C), 116.3 (C), 110.1 (CH), 68.2 (C), 65.3 (CH), 62.6 (C/CH_2), 48.3 (C/CH_2), 40.7 (CH_2), 23.3 (CH_3), 23.1 (CH_3), 20.6 (CH_3).

ESI-MS, positive mode: *m/z* (rel. int., %) = 371 (100) $[M+H]^+$.

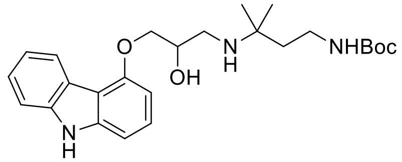

Compound 20 was prepared from 4-(glycidyloxy)carbazole (500 mg, 2.09 mmol) and 982 mg (4.12 mmol) of (3-amino-3-methylbutyl)carbamic acid *tert*-butyl ester (J & W PharmLab LLC) as described by Taylor et al.[9]

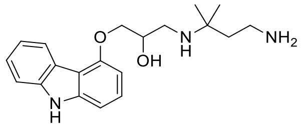

Compound 21 was prepared from **20** (271 mg, 0.61 mmol) and 0.3 ml 4 M HCl in 1,4-dioxane. The precipitate of the crude hydrochloride salt was filtered off and dried. The title compound was isolated by column chromatography on RP-C_{18} ($MeOH/H_2O$ 1:1 +0.1% NH_4OH). Yield – 200 mg (96%) of white solid.

^1H NMR (400 MHz, DMSO-d_6): δ = 11.33 (s, 1H, NH), 8.20 (d, *J* = 7.8, 1H), 7.42 (dt, *J* = 8.2, 0.9 Hz, 1H), 7.34 – 7.24 (m, 2H), 7.11 (ddd, *J* = 8.0, 7.2, 1.0, 1H), 7.05 (dd, *J* = 8.1, 0.6, 1H), 6.68 (dd, *J* = 8.1, 0.6, 1H), 4.15 (d, $^3J_{H,H}$ = 5.3, 2H, CH_2O), 4.08 – 4.01 (m, 1H, CHO), 2.88 – 2.82 (m, 3H, CH_2N, H^A from CH^AH^BN), 2.75 –2.68 (m, 1H, H^B from CH^AH^BN),1.63 (m, 2H, CH_2), 1.07 (s, 6H, CH_2).

^{13}C NMR (101 MHz, DMSO-d_6): δ = 154.9, 141.1, 138.9, 126.5, 124.5, 122.5, 121.7, 118.5, 111.6, 103.9, 100.4, 70.6, 69.0, 51.9, 44.9, 35.4, 26.5, 21.8.

ESI-MS, positive mode: *m/z* (rel. int., %) = 342 (100) $[M+H]^+$.

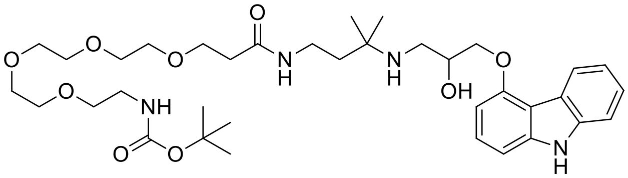

Compound 22. Amine **21** (70 mg, 0.21 mmol) was dissolved in 0.5 ml DMSO, and 112 mg (0.31 mmol) of 15-(Boc-amino)-4,7,10,13-tetraoxapentadecanoic acid in 0.3 ml DMSO was added. HATU (133 mg, 0.35 mmol) in 0.3 ml DMSO and 50 µl Et_3N were then added. The reaction mixture was stirred for one hour at room temperature. The solvents were evaporated *in vacuo*, and the product was isolated by flash chromatography (Biotage SNAP Ultra 10 g; gradient 3% to 65% MeOH in DCM). Yield – 78 mg (54%) of white solid.

^1H NMR (400 MHz, $CDCl_3$): δ = 8.51 (s, 1H, NH), 8.27 (ddt, *J* = 8.0, 1.3, 0.7, 1H), 7.45 – 7.35 (m, 2H), 7.32 (t, *J* = 8.0, 1H), 7.21 (ddd, *J* = 8.1, 7.0, 1.3, 1H), 7.07 (dd, *J* = 8.1, 0.7, 1H), 6.68 (dd, *J* = 8.0, 0.7, 1H), 4.36 – 4.29 (m, 1H, CHO), 4.28 – 4.21 (m, 2H, CH_2O), 3.69 – 3.49 (m, 14H, CH_2O), 3.45 (t, *J* = 5.2, 2H, CH_2N), 3.28 (dd, *J* = 13.0, 6.9, 4H, CH_2), 3.05 (dd, *J* = 12.0, 3.3, 1H, OH/NH), 2.91 (dd, *J* = 12.0, 6.7, 1H, NH/OH), 2.34 (t, *J* = 6.0, 2H, CH_2), 1.59 (t, *J* = 7.3, 2H, CH_2), 1.43 (s, 9H, CH_3), 1.14 (s, 6H, CH_3).

^{13}C NMR (101 MHz, $CDCl_3$): δ = 156.0, 155.0, 141.0, 138.8, 126.7, 125.0, 122.8, 122.4, 119.6, 112.6, 110.3, 110.1, 104.0, 101.2, 70.5, 70.4, 70.4, 70.3, 70.2, 70.1, 70.1, 69.3, 68.0, 67.4, 52.7, 44.8, 40.3, 39.0, 37.1, 35.7, 28.4, 27.2, 27.1.

ESI-MS, positive mode: *m/z* (rel. int., %) = 689 (100) $[M+H]^+$.

HRMS (*m/z*): $[M+H]^+$ calcd. for $C_{36}H_{56}N_4O_9$, 689.4120; found, 689.4107.

Compound 23. Boc-protected amine **22** (20 mg, 0.03 mmol) was dissolved in 1 ml DCM and the solution was cooled to 0 °C. The 1:1 mixture TFA/DCM (0.15 ml) was added, and the reaction mixture was stirred overnight. The solvents were evaporated *in vacuo*, and the product was isolated by flash chromatography (Biotage SNAP Ultra 10 g; gradient 3% to 65% of MeOH in DCM). Yield – 78 mg (54%) of white solid.

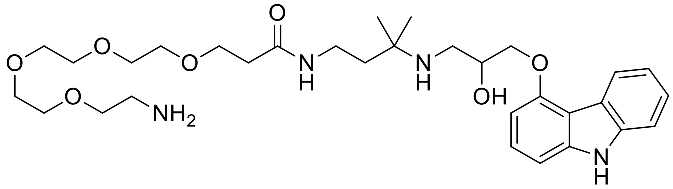

^{1}H NMR (400 MHz, CD_3OD): δ = 8.29 (dt, *J* = 7.8, 1.0, 1H), 7.41 (dt, *J* = 8.1, 1.0, 1H), 7.37 – 7.23 (m, 2H), 7.13 (ddd, *J* = 8.1, 7.1, 1.0, 1H), 7.06 (dd, *J* = 8.1, 0.7, 1H), 6.69 (dd, *J* = 8.0, 0.7, 1H), 4.31 – 4.17 (m, 3H, CH, CH_2), 3.65 (t, *J* = 6.1, 2H, CH_2), 3.56 – 3.50 (m, 12H, CH_2), 3.45 (t, *J* = 5.3, 2H, CH_2), 3.25 (t, *J* = 7.8, 2H, CH_2), 3.05 – 2.99 (m, 1H, CH_2), 2.90 – 2.83 (m, 1H, CH_2), 2.73 (t, *J* = 5.3, 2H, CH_2), 2.36 (t, *J* = 6.2, 2H, CH_2), 1.65 (td, *J* = 7.6, 5.1, 2H, CH_2), 1.16 (s, 3H, CH_3), 1.15 (s, 3H, CH_3).

^{13}C NMR (101 MHz, CD_3OD): δ = 156.3, 141.4, 136.6, 131.2, 127.5, 125.8, 123.5, 121.9, 119.8, 111.3, 110.2, 105.2, 101.6, 71.5, 71.4, 71.3, 71.3, 70.6, 69.8, 68.1, 68.1, 66.4, 55.7, 46.1, 43.7, 39.3, 37.7, 36.2, 29.9, 24.2.

ESI-MS, positive mode: *m/z* (rel. int., %) = 295 (100) $[M+2H]^{2+}$; 589 (10) $[M+H]^+$.

HRMS (*m/z*): $[M+H]^+$ calcd. for $C_{31}H_{48}N_4O_7$, 589.3596; found, 589.3606.

Compound 24

Dye KK114[8] (4 mg, 4.5 µmol) was suspended in 2 ml of MeCN (dry), then 10 µl Et_3N and 6 mg (10.2 µmol) of amine **23** in 1 ml MeCN (dry) were added. The reaction mixture was cooled to 0°C and the solution of HATU (10 mg, 26 µmol) in 2 mL MeCN (dry) was added. The reaction mixture was stirred for 2 h at room temperature. The title compound was isolated on RP-C_{18} (50 mL) using MeCN/H_2O 1:1 + 0.1% Et_3N mixture as an eluent. Yield – 1.5 mg (23%) of blue solid. HPLC: t_R = 9.5 min, (A/B: 20/80 – 100/0 in 20 min, 1.2 ml/min, 636 nm).

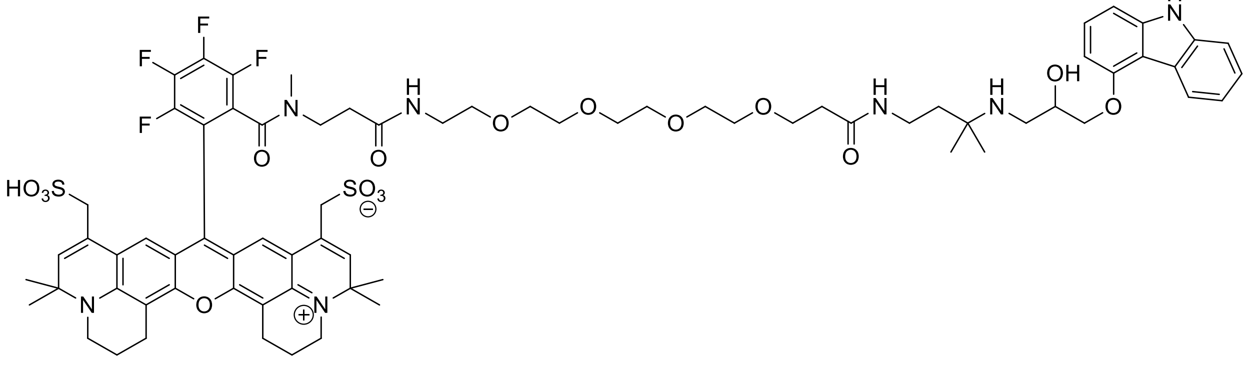

^{1}H NMR (500 MHz, CD_3OD): δ = 8.10 – 8.04 (m, 1H), 7.40 – 7.32 (m, 2H), 7.28 (d, J = 7.5, 1H), 7.21 (t, J = 7.5, 1H), 7.17 (t, J = 7.9, 1H), 7.06 – 6.94 (m, 2H), 6.58 (d, J = 7.7, 1H), 5.86 – 5.80 (m, 2H), 4.34 (br.s, 1H, CH), 4.26 – 4.19 (m, 2H, CH_2), 4.18 – 4.07 (m, 2H, CH_2), 3.85 – 3.72 (m, 2H, CH_2), 3.67 (m, 2H, CH_2), 3.57 – 3.47 (m, 16H, CH_2), 3.47 – 3.42 (m, 2H, CH_2), 3.38 (t, J = 5.5 Hz, 2H, CH_2), 3.36 – 3.27 (m, 2H, CH_2), 3.26 – 3.19 (m, 2H, CH_2), 2.86 (m, 3H, CH_3), 2.71 (s, 2H, CH_2), 2.67 – 2.56 (m, 2H, CH_2), 2.40 (t, J = 6.1, 2H, CH_2), 2.20 – 2.11 (m, 2H, CH_2), 1.97 – 1.87 (m, 6H, MeC=CH), 1.84 (s, 2H, CH_2), 1.55 – 1.46 (m, 12H, CH_3), 1.42 (s, 3H, CH_3), (1.41 (s, 3H, CH_3).

^{13}C NMR (126 MHz, CD_3OD; highly split signals of the fluorinated ^{13}C-atoms were not registered): δ = 174.4, 172.8, 163.0, 155.9, 154.5, 152.2, 151.9, 143.0, 142.9, 140.6, 138.9, 138.81, 138.75, 138.1, 138.0, 127.4, 125.8, 125.4, 124.8, 124.6, 123.5, 123.2, 122.7, 122.5, 121.6, 119.9, 114.3, 113.4, 111.4, 107.6, 106.8, 105.5, 101.5, 71.4, 71.4, 71.3, 71.2, 70.7, 70.3, 68.1, 67.3, 61.5, 61.4, 61.3, 61.2, 60.1, 55.1, 54.9, 45.9, 45.5, 44.5, 40.3, 38.3, 38.1, 37.6, 35.6, 34.2, 28.8, 28.5, 28.3, 23.84, 23.80, 21.4, 21.3, 20.9, 18.7, 17.3.

ESI-MS, positive mode: *m/z* (rel. int., %) = 1457.3 (100) $[M\text{-H}]^-$.

HRMS (*m/z*): $[M+H]^+$ calcd. for $C_{73}H_{87}N_7O_{16}S_2$, 1458.5660; found, 1458.5671.

Supplementary Results

Supplementary Tables

Supplementary Table 1. Spectral properties of the dyes of the present study.

Dye	Absorption		Emission		τ^a, ns
	λ_{max}, nm	ε, M^{-1} m^{-1}	λ_{max}, nm	Φ_{fl}	
BODIPY 630-X[b]	625	101000	640	0.76	3.9
KK114[c]	637	92000	660	0.55	3.6
610CP[d]	609	100000	634	0.59	3.1

[a]τ - fluorescence lifetime; [b]in MeOH; [c]in H_2O, see ref. [8]; [d]in aqueous PBS buffer (pH 7.4).

Supplementary Table 2. Properties of fluorescent β$_2$AR ligands

Probe	Absorption/ Emission (nm)	Fluorescence increase[a]	Linker length (nm)[b]
		BI-106107 derivatives	
BI-4C-610CP (**16**)	613/635	1.5±0.4	1.1
BI-PEG-BDY630 (**17**)	634/646	10.5±1.7	2.4
BI-PEG-KK114 (**18**)	638/656	1.4±0.1	2.0
BI-PEG-610CP (**19**)	613/635	1.2±0.3	1.6
		Carazolol derivatives	
ab118171	634/644	8.2±3.9	1.6
Carazolol-KK114 (**24**)	642/656	5.9±1.5	2.7

[a]upon addition of 0.1% SDS to a solution of the corresponding ligand in PBS (pH 7.4) with 1 mg/ml bovine serum albumin (BSA). Values are mean ± SD, n=3 independent measurements; [b]the linker length is calculated: for agonists - starting from the position 3 in the *o*-tolyl residue of BI-167107 fragment and ending at the attachment point of the dye (6'-carboxamide group in the pendant phenyl ring of 610CP dye and N-methylcarboxamide group in the o-position of the pendant phenyl ring of KK114 dye). Note that the molecule of BODIPY 630/650-X has an additional aliphatic linker of 3.8 nm length. For antagonists - from the isopropyl fragment of carazolol and ending at the attachment point of the dye.

Supplementary Table 3. Quantum yields in PBS pH 7.4 buffer

Sample name	Dye	
	KK114	BODIPY 630/650
Dye alone	0.50	0.44
1 µM dye + 1 mM carazolol	0.23	0.14
1 µM dye + 1 mM BI-167107	0.50	0.35
carazolol probe	0.16	n.d.[a]
BI-167107 probe	0.52	0.09

[a] n.d. – not determined

Supplementary Table 4. Fitted full width at half maximum (FWHM) value of profiles shown in figure 7

Figure name	$FWHM_{Confocal}$	$FWHM_{STED}$	$FWHM_{Confocal}$ / $FWHM_{STED}$
Figure 7b	300 ± 8 nm	125 ± 7 nm	2.4
Figure 7d	188 ± 40 nm & 172 ± 49 nm	101 ± 40 nm & 108 ± 16 nm	1.9 & 1.6

Supplementary Figures

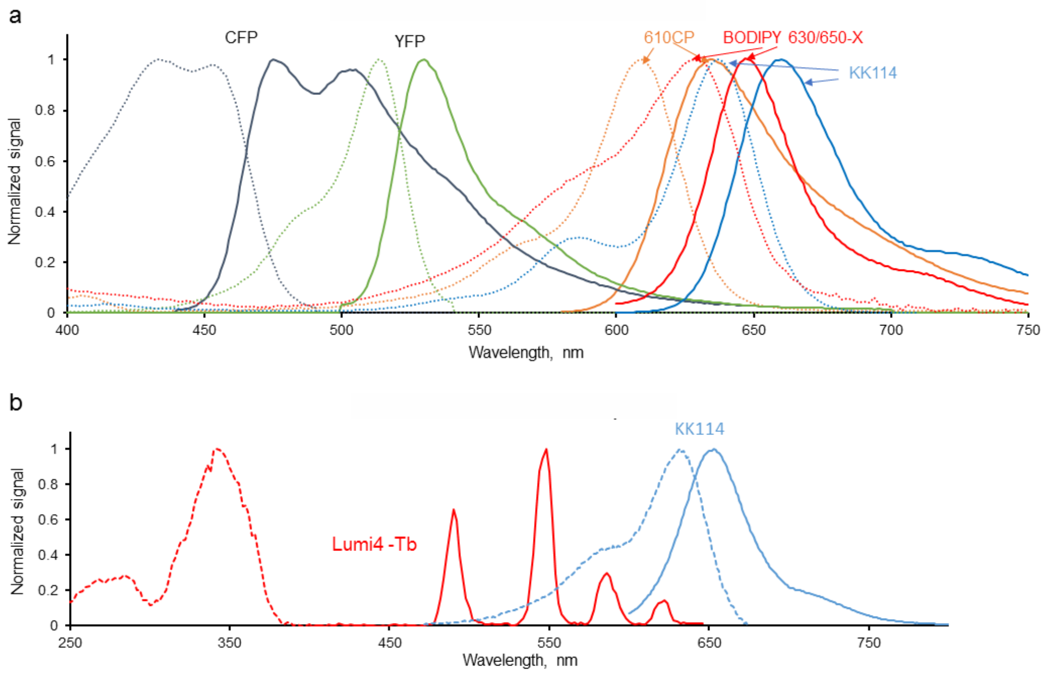

Supplementary Figure 1. Spectra of fluorophores used in this study. Excitation (dashed lines) and emission (solid lines) spectra of (a) cyan and yellow fluorescent proteins and dyes used in the study; (b) Tb^{3+} cryptate (Lumi4-Tb) as the donor fluorophore and fluorescent dye KK114 as the acceptor fluorophore used in time resolved fluorescence energy transfer experiment (K_d determination). The spectra are averaged from three measurements.

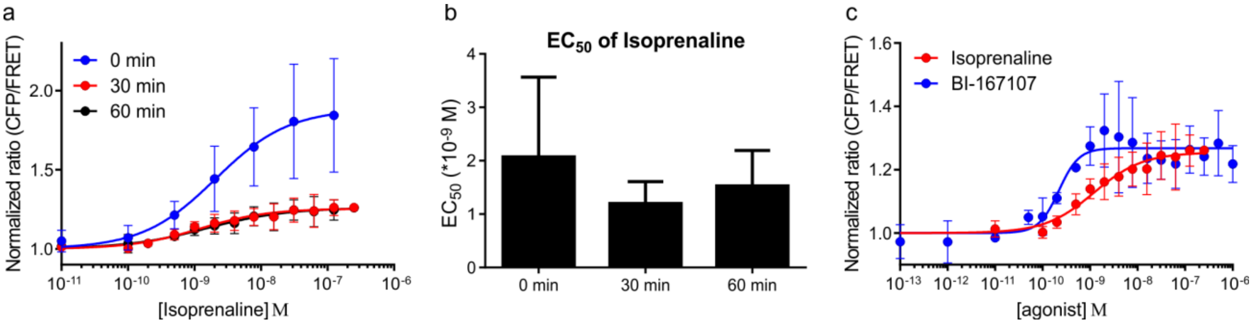

Supplementary Figure 2. Intracellular cAMP sensor response to β-AR agonists. (a) dose-response curves for isoprenaline at different time points; (b) fitted isoprenaline EC_{50} at different time points; (c) comparison of cAMP sensor response after 30 min incubation with either isoprenaline or BI-167107. HEK 293 cells expressing 3',5'-cAMP FRET sensor were treated with increasing concentrations of isoprenaline or BI-167107. *R(CFP/FRET)* ratio was normalized to the *R(CFP/FRET)* of DMSO sample. Values presented as mean ± SD, n = 3 independent experiments.

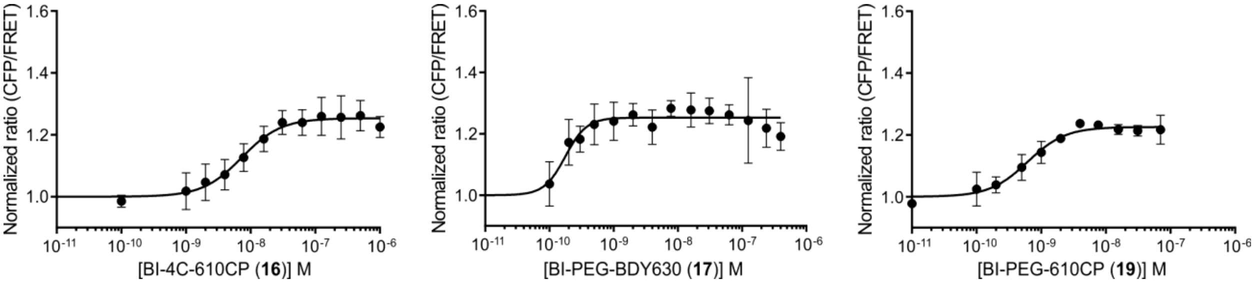

Supplementary Figure 3. Dose response curves of βAR agonist based probes in HEK 293 cells expressing 3',5'-cAMP FRET sensor. The cells were treated with increasing concentrations of BI-167107 fluorescent analogs. Sensor response *R(CFP/FRET)* was normalized to the *R(CFP/FRET)* of DMSO sample. The data correspond to 30 min incubation time and represent as mean ± SD, n = 2 for BI-5C-610CP or n = 3 for all other ligands.

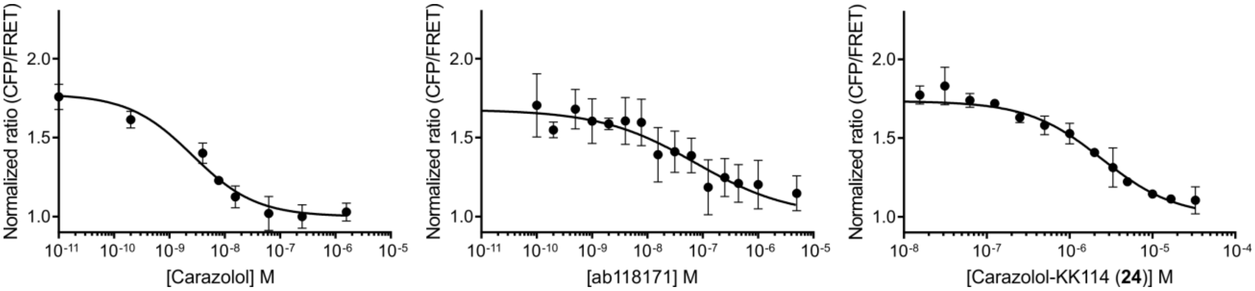

Supplementary Figure 4. Intracellular cAMP sensor response to competitive displacement of isoprenaline with βAR antagonists in HEK293 cells expressing biosensor ^{T}Epac^{VV}. The cells were treated with 17 nM isoprenaline and increasing concentrations of βAR antagonist. Fluorescence readout was performed after addition of the ligands - the time point “0 min”. Sensor response *R(CFP/FRET)* was normalized to the *R(CFP/FRET)* of DMSO sample. The data shown represent the mean ± SD, n = 3 independent experiments.

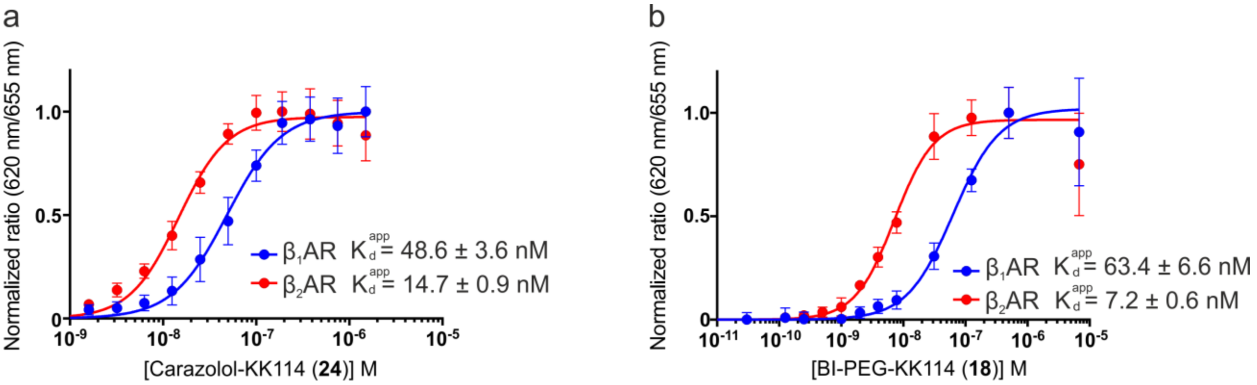

Supplementary Figure 5. **Saturation binding assay on cells expressing β_1ARs or β_2ARs labelled with Lumi4-Tb cryptate.** (a) β_1AR and β_2AR titration curves for carazolol-KK114 (**24**); (b) β_1AR and β_2AR titration curves for BI-PEG-KK114 (**18**). Data points are mean ± SD, n = 4 independent experiments. Note that the K_d^{app} fittings displayed Hill slope h > 1 (range of 1.2 - 1.6) indicating multiple βAR binding sites with positive cooperativity. K_d^{app} is presented as fitted mean ± SD.

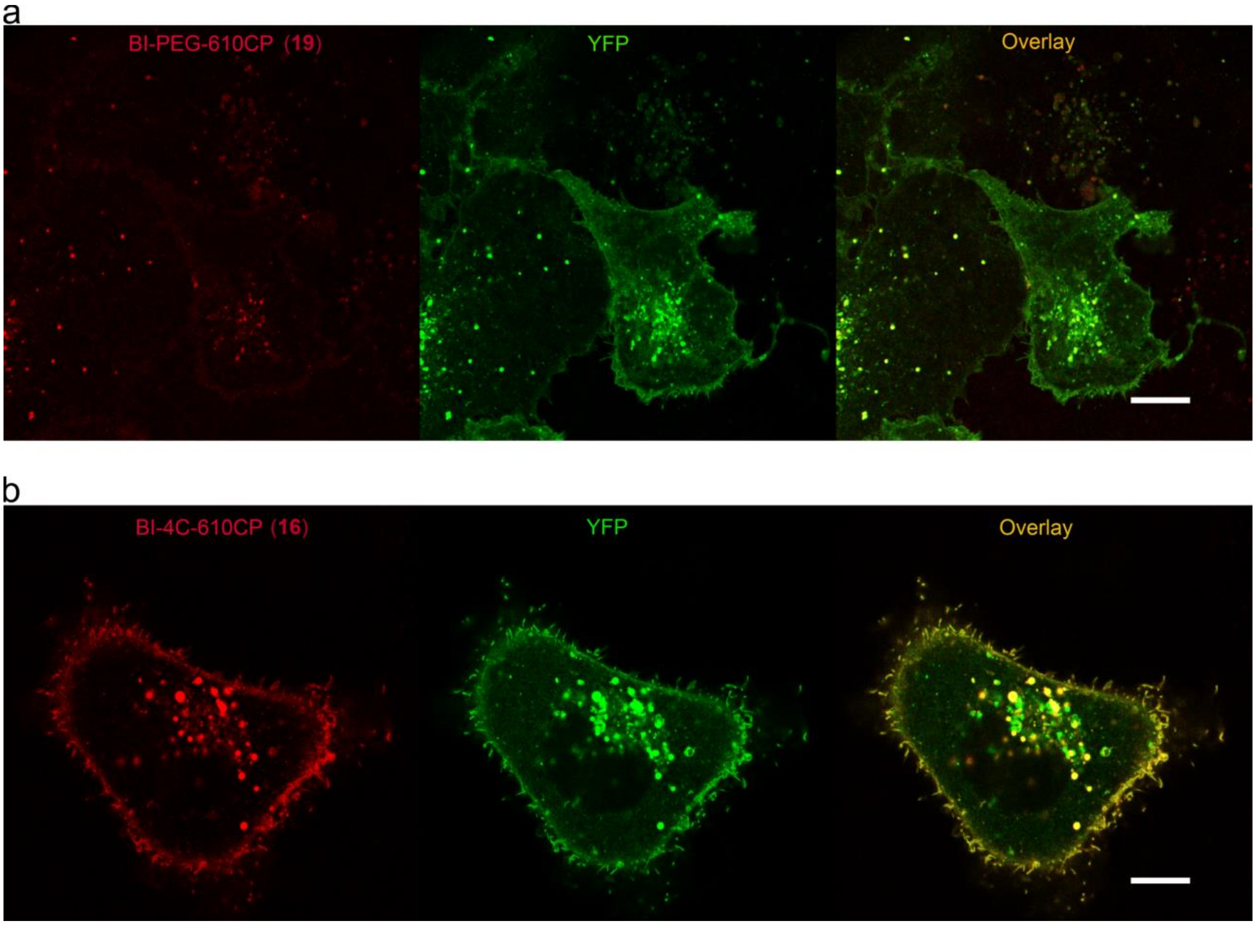

Supplementary Figure 6. Confocal images of live U2OS cells expressing β_2AR-YFP fusion protein and stained with 610CP ligands: (a) 1.9 µM BI-PEG-610CP (**19**), (b) 2.5 µM BI-4C-610CP (**16**). The cells were incubated in growth medium for 40 min at room temperature and washed two times with HBSS before imaging on a Leica SP8 inverted confocal microscope. The individual color channels and the overlays of both channels are shown. Scale bars 10 µm.

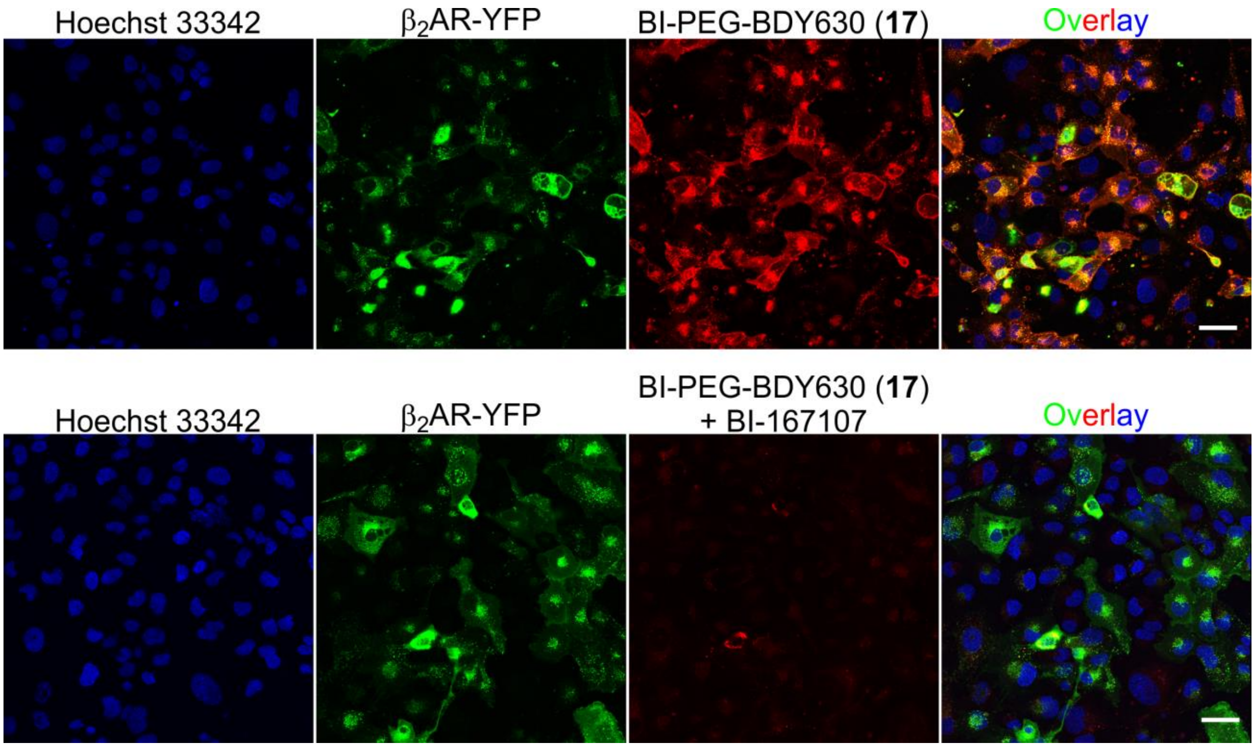

Supplementary Figure 7. Competitive binding experiment: BI-PEG-BDY630 (17). Living U2OS cells expressing β2AR-YFP fusion protein were incubated with a mixture of 100 nM BI-PEG-BDY630 (**17**) and 10 µM BI-167107 in growth medium in presence of 1 µg / ml Hoechst 33342 for 30 min and washed two times with HBSS before imaging on a Leica SP8 inverted confocal microscope. The individual color channels and their overlays are shown. Scale bars 50 µm.

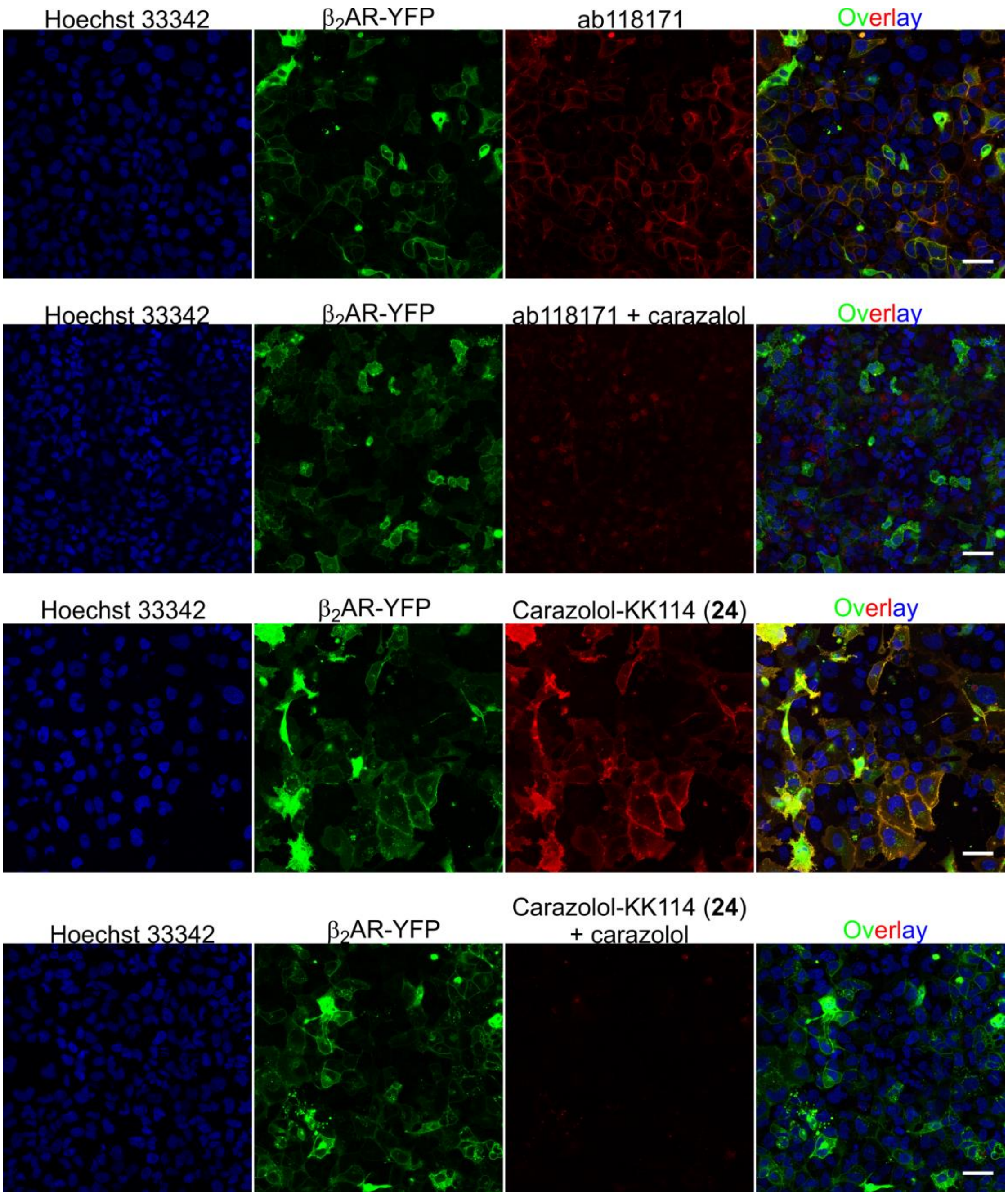

Supplementary Figure 8. Competitive displacement of ab118171 and carazolol KK114 (24) with parent ligand - carazolol. Living U2OS cells expressing β2AR-YFP fusion protein were treated with a mixture of 5 nM ab118171 and 10 µM carazolol or 100 nM carazolol-KK114 (**24**) and 10 µM carazolol. The cells were incubated in growth medium in presence of 0.1 µg / ml Hoechst 33342 for 30 min and washed two times before imaging on a Leica SP8 inverted confocal microscope. The individual color channels and their overlays are shown. Scale bars 50 µm.

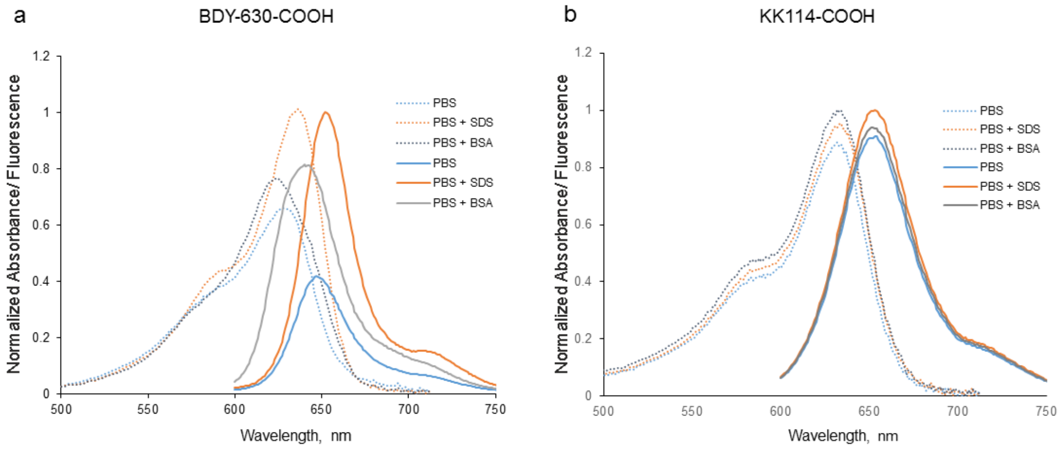

Supplementary Figure 9. Excitation and emission spectra of fluorescent dyes under different buffer conditions. (a) BODIPY 630/650 carboxylic acid and (b) KK114 carboxylic acid in PBS 7.4 without additives (gray), in the presence of 0.1% sodium dodecyl sulfate (SDS; orange) and in the presence of 0.1% bovine serum albumin (BSA; blue). Excitation (dashed lines) and emission (solid lines) spectra are averaged from three independent measurements.

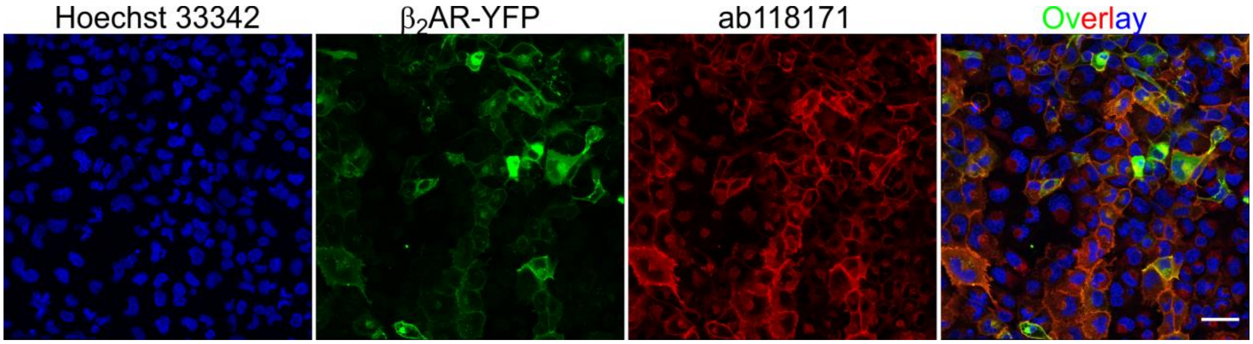

Supplementary Figure 10. No-wash images of living U2OS cells expressing β$_2$AR-YFP fusion protein. The cells were incubated with 5 nM ab118171 for 90 min in growth medium in the presence of 0.1 µg / ml Hoechst 33342 for 90 min and imaged on a Leica SP8 inverted confocal microscope without washing off the excess of probe. Scale bar 50 µm.

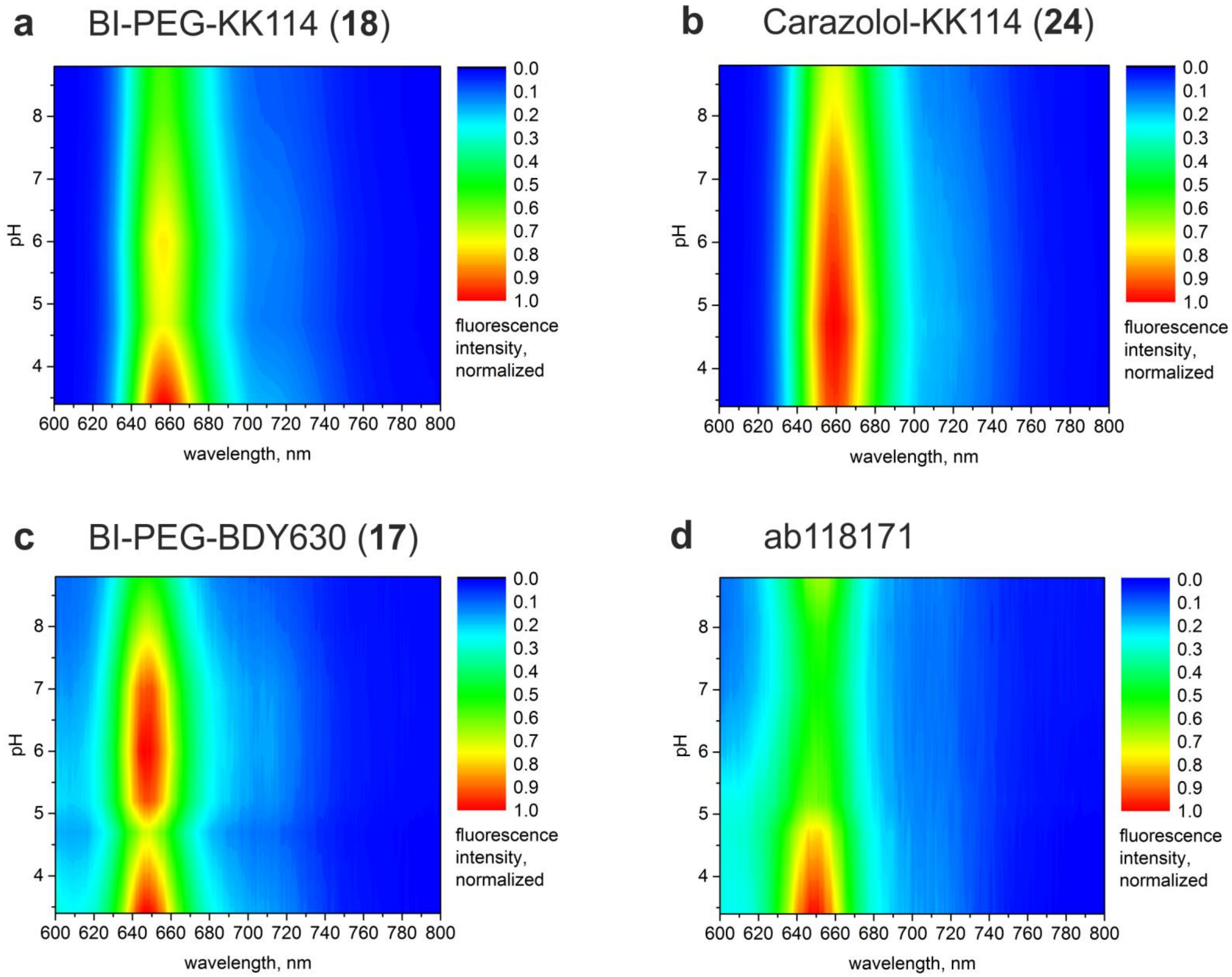

Supplementary Figure 11. Fluorescence emission spectra of the probes in phosphate buffer at different pH values. Fluorescence emission spectra of the probes (a) BI-PEG-KK114 (**18**); (b) carazolol-KK114 (**24**); (c) BI-PEG-BDY630 (**17**) and (d) ab118171 in 0.2 M Na-phosphate buffer (pH 4.7 – 8.0, + 1 v/v% DMSO). The spectra have been recorded in triplicate in black polystyrene 96 well plates (flat bottom) on a Spark 20M (Tecan) microplate reader at 25 °C. The background is normalized to DMSO.

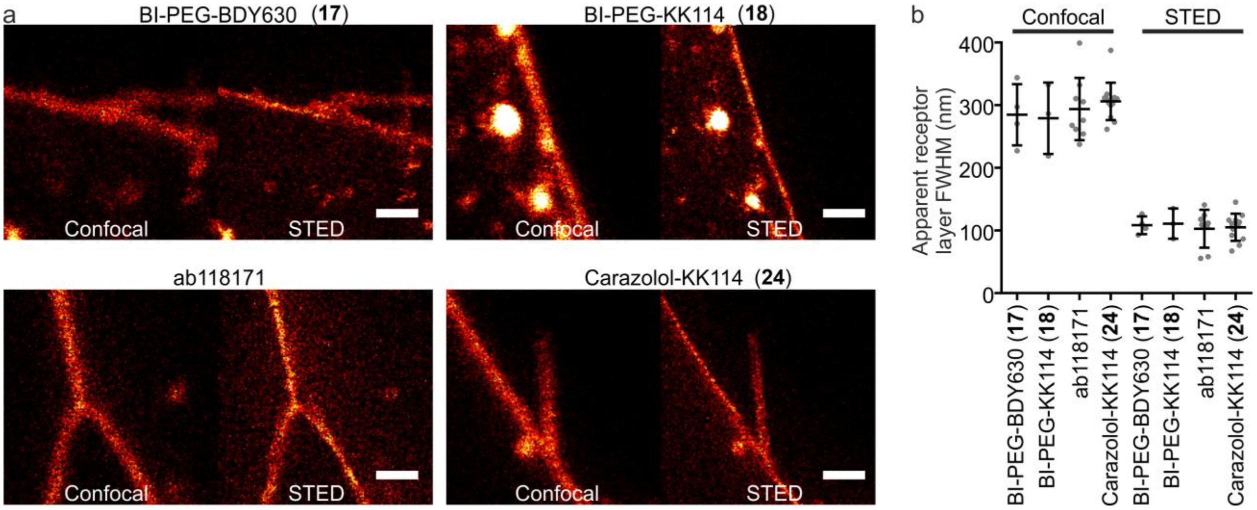

Supplementary Figure 12. Evaluation of confocal and STED images of living U2OS cells expressing β$_2$AR-YFP stained with fluorescent ligands. (a) Examples of confocal and STED images of the cells incubated for 30–40 min in growth medium in a presence of fluorescent ligand (100 nM for BI-PEG-BDY630 (**17**), BI-PEG-KK114 (**18**), carazolol-KK114 (**24**) and 5 nM for ab118171) and imaged on an Abberior STED 775 QUAD scanning microscope. Excitation with a 640 nm diode laser (10 mW). Detection at 670 ± 40 nm, pulsed STED at 760 nm with power of 40 mW at the back aperture of the objective; pulse duration 300 ps at 76 MHz repetition rate. Scale bars 1 µm. The apparent receptor layer thickness: confocal ~270 nm; STED ~100 nm. (b) The apparent receptor layer thickness determined by confocal and STED imaging. Every dot indicates a single measurement of the apparent receptor layer thickness in the different areas of the image, images of at least 3 cells were taken for the determinations. The error bars represent ± SD.

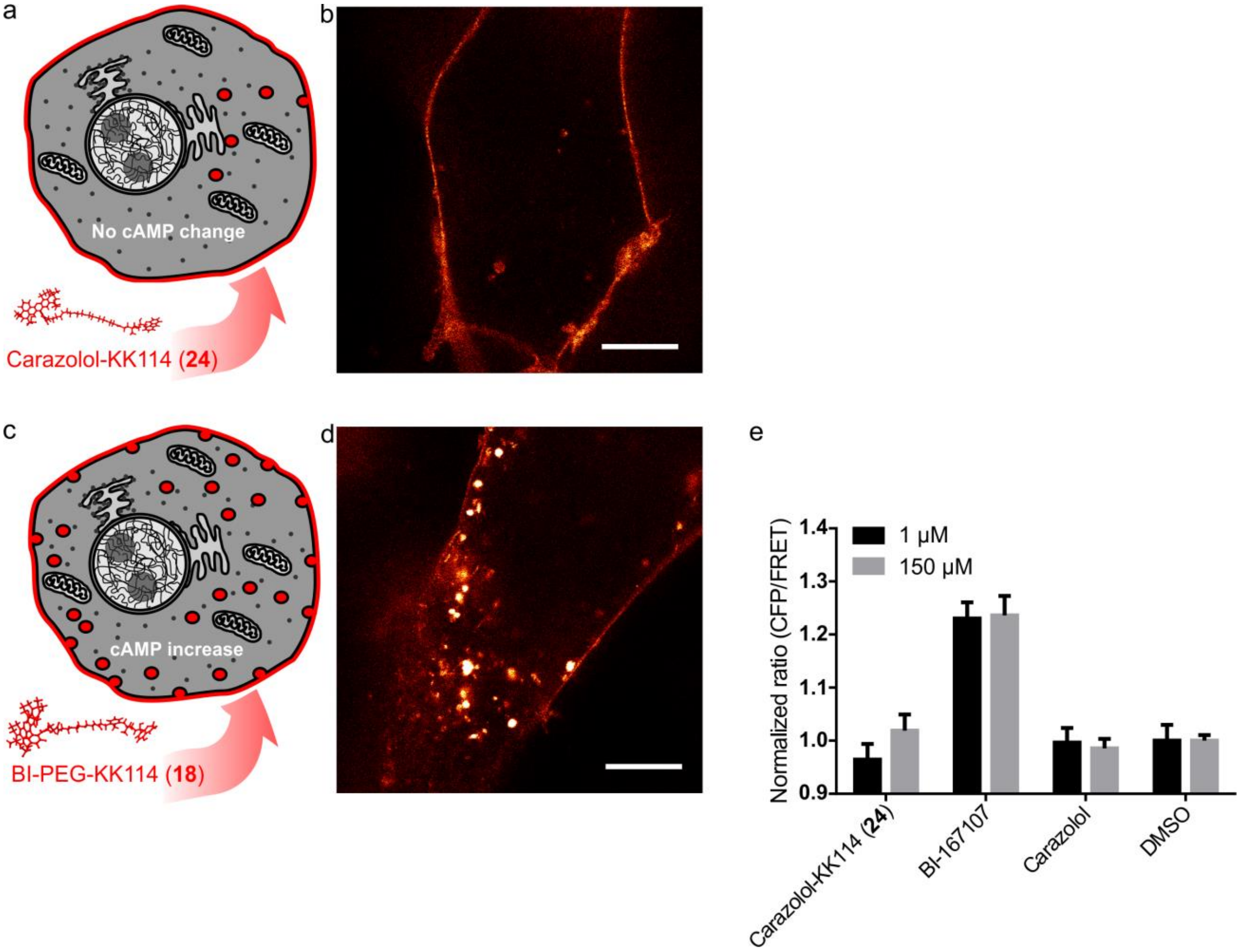

Supplementary Figure 13 Summary of fluorescent ligands properties used for staining of living cells. (a) Binding of carazolol-KK114 (**24**) to the βARs does not induce increase of intracellular cAMP levels and extensive receptor internalization. (**b**) STED image of U2OS cell expressing β_2AR-YFP and stained with 100 nM carazolol-KK114 (**24**). The cells were incubated for 40 min in growth medium in the presence of 100 nM carazolol-KK114 (**24**) and washed two times before imaging on an Abberior STED 775 QUAD scanning microscope. Scale bar 5 µm; (**c**) Binding of BI-PEG-KK114 (**18**) to the βAR induces intracellular cAMP level increase and extensive internalization of receptors. (**d**) STED image of U2OS cells expressing β_2AR-YFP and stained with 100 nM BI-PEG-KK114 (**18**). The cells were incubated for 40 min in growth medium in the presence of 100 nM BI-PEG-KK114 (**18**) and washed two times before imaging on an Abberior STED 775 QUAD scanning microscope. Excitation with a 640 nm diode laser (100 mW). Detection at 670 ± 40 nm, pulsed STED at 760 nm with power of 40 mW at the back aperture of the objective; pulse duration 300 ps at 76 MHz repetition rate. Scale bar 5 µm; (**e**) cAMP production response in HEK 293 cells expressing 3',5'-cAMP FRET sensor cells**.** Cells were incubated with compounds for 60 min at room temperature before measurements. Data presented as mean ± SD, n=2 independent measurements.

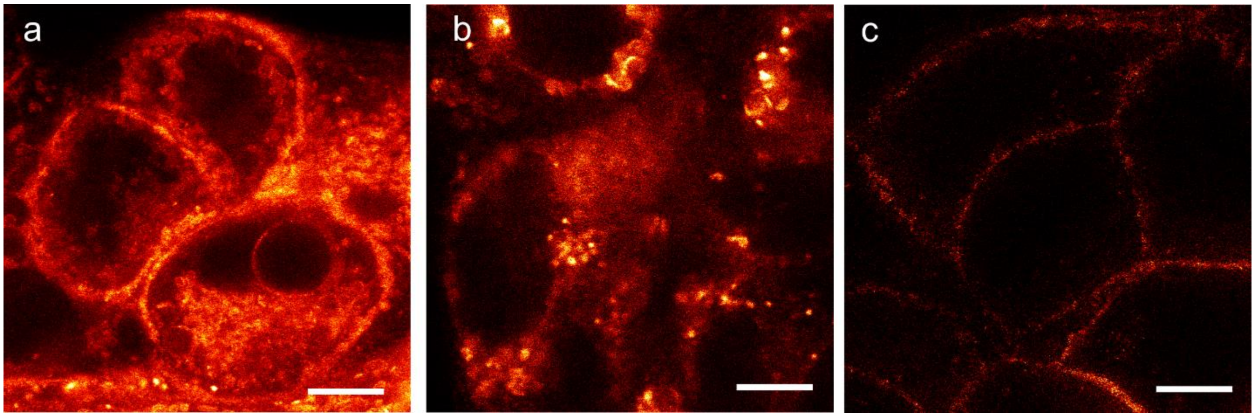

Supplementary Figure 14. STED images of living CAPAN-1 cells stained with fluorescent ligands. The cells were incubated for 30–40 min in growth medium in the presence of a fluorescent ligand: (a) 100 nM BI-PEG-BDY630 (**17**); (b) 10 nM ab118171; (c) 100 nM carazolol-KK114 (**24**) and washed two times before imaging on an Abberior STED 775 QUAD scanning microscope. Excitation with a 640 nm diode laser (100 mW). Detection at 670 ± 40 nm, pulsed STED at 760 nm with power of 40 mW at the back aperture of the objective; pulse duration 300 ps at 76 MHz repetition rate. Scale bars 5 µm.

Fiji image processing script

```
selectImage(1);
dir = getDirectory ("image");
name = getTitle();
dot = indexOf(name,".lif -");
OriginalName = substring(name, 0, dot);
getDimensions(x,y,c,z,t);
run("Make Composite", "display=Composite");
Stack.setDisplayMode("color");
Stack.setChannel(1);
run("Grays");
call("ij.ImagePlus.setDefault16bitRange", 0);
setMinAndMax(0, 1500);
Stack.setChannel(2);
run("Grays");
call("ij.ImagePlus.setDefault16bitRange", 0);
setMinAndMax(0, 4000);
Stack.setChannel(3);
run("Grays");
call("ij.ImagePlus.setDefault16bitRange", 0);
setMinAndMax(0, 4000);
Stack.setDisplayMode("composite");
run("Make Montage...", "columns=3 rows=1 scale=1 first=1 last=3");
selectImage(1);
Stack.setDisplayMode("color");
Stack.setChannel(1);
run("Blue");
Stack.setChannel(2);
run("Green");
Stack.setChannel(3);
run("Red");
Stack.setDisplayMode("composite");
run("Stack to RGB");
run("Combine...");
run("Scale Bar...", "width=50 height=50 font=56 color=White background=None location=[Lower Right]
bold hide");
run("downsample", "width=4000 height=1000 source=0.50 target=0.50 keep");
selectImage(1);
saveAs("Tiff", ""+dir+OriginalName+"_Composite.tif");
run("Stack to RGB");
saveAs("Tiff", ""+dir+OriginalName+"_RGB.tif")
close();
saveAs("Tiff", ""+dir+OriginalName+"_montage_RGB.tif")
run("Close All");
```

Supplementary abbreviations

βAR, β adrenergic receptors; β_2AR, β_2 adrenergic receptors; BDB, 5-(benzyloxy)-8-(dihydroxyacetyl)-2*H*-1,4-benzoxazin-3(4*H*)-one; Boc, *N*-*tert*-butoxycarbonyl group; BDY 630 or BODIPY 630/650, 4,4-difluoro-5-(2-thienyl)-3a,4a-diaza-4-bora-s-indacen-3-yl-ethenylbenzene; Cbz, *N*-benzyloxycarbonyl group; DCM, dichloromethane; DIEA, *N*,*N*-diisopropylethylamine; Epac, exchange protein activated by cAMP; Et_2O, diethyl ether; EtOAc, ethyl acetate; FRET, fluorescence resonance energy transfer; GFP, green fluorescent protein; HATU, *O*-(7-azabenzotriazol-1-yl)-*N*,*N*,*N*′,*N*′-tetramethyluroniumhexafluorphosphate; HBSS, Hank's balanced salt solution; HEK 293, human embryonic kidney 293 cells; Hex, hexane; IA, intrinsic activitiy; K_d, dissociation constant; NaHMDS, sodium bis(trimethylsilyl)amide; NHS, *N*-hydroxysuccinimide; $Pd(dba)_2$, bis(dibenzylideneacetone)palladium(0); $Pd(dppf)Cl_2$, [1,1'-bis(diphenylphosphino)ferrocene]palladium(II) dichloride; PyBOP, benzotriazol-1-yl-oxytripyrrolidinophosphonium hexafluorophosphate; SPhos, 2-dicyclohexylphosphino-2′,6′-dimethoxybiphenyl; RP-HPLC, reverse phase high-performance liquid chromatography; STED, stimulated emission depletion microscopy; TFA, trifluoroacetic acid; TLC, thin-layer chromatography; U2OS, human osteosarcoma cells; YFP, yellow fluorescent protein.

Supplementary references

1 Bonnert, R. V. & Connolly, S. Preparation of benzoxazinone derivatives for use in combination chemotherapy for treatment of respiratory diseases. WO2011073662A1 (2011).

2 Floyd, M. B., Du, M. T., Fabio, P. F., Jacob, L. A. & Johnson, B. D. The Oxidation of Acetophenones to Arylglyoxals with Aqueous Hydrobromic Acid in Dimethylsulfoxide. *J Org Chem* **50**, 5022-5027 (1985).

3 Winum, J. Y., Kamal, M., Leydet, A., Rogue, J. P. & Montero, J. L. Homologation of carboxylic acids by Arndt-Eistert reaction under ultrasonic waves. *Tetrahedron Lett* **37**, 1781-1782 (1996).

4 Glossop, P. A., Lane, C. A. L., Lunn, G. & Price, D. A. N-Arylalkyl amides and related derivatives as β2 antagonists, and their preparation, pharmaceutical compositions, and use for the treatment of inflammatory, allergic and respiratory diseases. WO2006051373A1 (2006).

5 Pearson, A. J. & Heo, J. N. Synthetic studies on the DEF ring system of ristocetin A via ruthenium-promoted SNAr reaction: problems and solutions using arylserine-Ru complexes. *Tetrahedron Lett* **41**, 5991-5996 (2000).

6 Roy, B. C. & Mallik, S. Synthesis of new polymerizable metal-chelating lipids. *J Org Chem* **64**, 2969-2974 (1999).

7 Butkevich, A. N. *et al.* Fluorescent Rhodamines and Fluorogenic Carbopyronines for Super-Resolution STED Microscopy in Living Cells. *Angew Chem Int Edit* **55**, 3290-3294 (2016).

8 Kolmakov, K. *et al.* Red-Emitting Rhodamine Dyes for Fluorescence Microscopy and Nanoscopy. *Chem-Eur J* **16**, 158-166 (2010).

9 Taylor, M. G., Klenke, B., Suzdak, P. D. & Mazhari, R. Preparation of oxopyridazinyl derivatives with inhibitory activity against beta-adrenergic receptors and phosphodiesterase. WO2006060122A2 (2006).
